# Ddp1 Cooperates with Ppx1 to Counter a Stress Response Initiated by Nonvacuolar Polyphosphate

**DOI:** 10.1128/mbio.00390-22

**Published:** 2022-07-07

**Authors:** Liam McCarthy, Iryna Abramchuk, Gamal Wafy, Alix Denoncourt, Mathieu Lavallée-Adam, Michael Downey

**Affiliations:** a Department of Cellular and Molecular Medicine, University of Ottawa, Ottawa, Ontario, Canada; b Ottawa Institute of Systems Biology, University of Ottawa, Ottawa, Ontario, Canada; c Department of Biochemistry, Microbiology and Immunology, University of Ottawa, Ottawa, Ontario, Canada; Newcastle University; Tel Aviv University

**Keywords:** polyphosphate, proteomics, polyP, stress response, Msn2, PPK, Vtc4, Hog1, Yak1, PKA, Ppx1, stress, vacuole, yeast

## Abstract

In diverse cells from bacterial to mammalian species, inorganic phosphate is stored in long chains called polyphosphate (polyP). These nearly universal polymers, ranging from three to thousands of phosphate moieties in length, are associated with molecular functions, including energy homeostasis, protein folding, and cell signaling. In many cell types, polyphosphate is concentrated in subcellular compartments or organelles. In the budding yeast Saccharomyces cerevisiae, polyP synthesis by the membrane-bound vacuolar transporter chaperone (VTC) complex is coupled to its translocation into the lumen of the vacuole, a lysosome-like organelle, where it is stored at high concentrations. In contrast, the ectopic expression of the bacterial polyphosphate kinase (PPK) results in the toxic accumulation of polyP outside the vacuole. In this study, we used label-free mass spectrometry to investigate the mechanisms underlying this toxicity. We find that PPK expression results in the activation of a stress response mediated in part by the Hog1 and Yak1 kinases and the Msn2/Msn4 transcription factors as well as by changes in protein kinase A (PKA) activity. This response is countered by the combined action of the Ddp1 and Ppx1 polyphosphatases that function together to counter polyP accumulation and downstream toxicity. In contrast, the ectopic expression of previously proposed mammalian polyphosphatases did not impact PPK-mediated toxicity in this model, suggesting either that these enzymes do not function directly as polyphosphatases *in vivo* or that they require cofactors unique to higher eukaryotes. Our work provides insight into why polyP accumulation outside lysosome-like organelles is toxic. Furthermore, it serves as a resource for exploring how polyP may impact conserved biological processes at a molecular level.

## INTRODUCTION

Inorganic phosphates can be joined in linear chains that range from three to thousands of residues in length. These polymers, called polyphosphate, or “polyP,” have been implicated in diverse biological processes spanning both prokaryotic and eukaryotic species. In the budding yeast Saccharomyces cerevisiae, polyP is synthesized by the vacuolar transporter chaperone (VTC) complex (1). The VTC complex is composed of the catalytic subunit Vtc4 along with Vtc1 and either Vtc2 or Vtc3 (1). Cells mutated for Vtc4, Vtc1, or both Vtc2 and Vtc3 have no detectable polyphosphate (2). An accessory subunit called Vtc5 increases the activity of the complex but is not strictly required for polyP synthesis (3, 4). The majority of the VTC complex localizes to the membrane of the yeast vacuole, a lysosome-like organelle (1). The VTC complex spans the vacuolar membrane and synthesizes polyP chains by hydrolyzing the gamma phosphate of cytoplasmic ATP, adding it to growing polyP chains that are simultaneously translocated into the vacuole lumen (5). Vacuolar polyP comprises the majority of total cellular polyP stores, exceeding 10 to 20% of the dry cell weight (2, 6). Other areas of the cell beyond the vacuole, including the plasma membrane, cytoplasm, and mitochondria, have lower levels of polyP (2). PolyP is also found in the nucleus, although the reported levels vary drastically (2). These variations are likely due to differences in growth conditions as well as differences in the methods of sample preparation and detection. Whether polyP outside the vacuole is synthesized locally or somehow transported from polyP stores that first accumulate in the vacuole lumen is unknown. Although some VTC proteins localize in part to the plasma membrane and/or the endoplasmic reticulum (1, 7), it is unknown if these pools of VTC proteins participate in polyP synthesis.

The subcellular enrichment of polyP is conserved in higher eukaryotes (8). For example, in human myeloma cells (9) and various cancer cell lines treated with cisplatin (10), polyP accumulates in the nucleolus. In platelets, it is concentrated in lysosome-related organelles called dense granules (11). The localization of polyP to such hot spots likely promotes specific functions. In yeast, vacuolar polyP is important for phosphate homeostasis (12) and vacuole fusion (13). PolyP released from dense granules following injury interacts directly with specific clotting factors to promote blood coagulation (11, 14). In contrast, less is known about whether there are physiological reasons why the levels of polyP in some areas of the cell must be kept low.

Unlike the VTC complex, the ectopic expression of the Escherichia coli polyphosphate kinase (called PPK) in yeast results in the accumulation of polyP outside the vacuole, and this correlates with slow growth, altered cellular morphology, and dramatic sensitivity to the translation inhibitor cycloheximide and the mTOR inhibitor rapamycin (5, 15). The amount of polyP made by PPK is 20% of that produced by VTC (5), and PPK-induced toxicity is observed in both wild-type (WT) and *vtc4*Δ cells (15). Together, these data point to the location of PPK-synthesized polyP, rather than changes in the total amount, as the driving force behind the observed toxicity. Finding the mechanisms underlying this toxicity will provide a foundation for a better understanding of the spatial and temporal regulation of polyP metabolism in eukaryotic cells.

Here, we use label-free mass spectrometry (MS) to determine why PPK expression and the concomitant accumulation of nonvacuolar polyP are toxic in yeast. Our results demonstrate that PPK-expressing cells remodel their proteome with changes to protein networks controlling fundamental aspects of metabolism. In follow-up work, we present evidence that a Yak1/Hog1-Msn2/Msn4 pathway limits the viability of these cells and that protein kinase A (PKA) activity is also altered by PPK expression. Finally, we demonstrate that polyP accumulation outside the vacuole is countered by the combined activities of the endopolyphosphatase Ddp1 (diphosphoinositol polyphosphate phosphohydrolase) and the exopolyphosphatase Ppx1 that work together to degrade long-chain polyP synthesized by PPK. Our results provide an explanation for why polyP is sequestered, elaborate functions of polyphosphatases outside the vacuole, and suggest new avenues for exploring polyP functions at the molecular level.

## RESULTS

### Remodeling of the yeast proteome in response to ectopic PPK expression.

**FIG 1:**
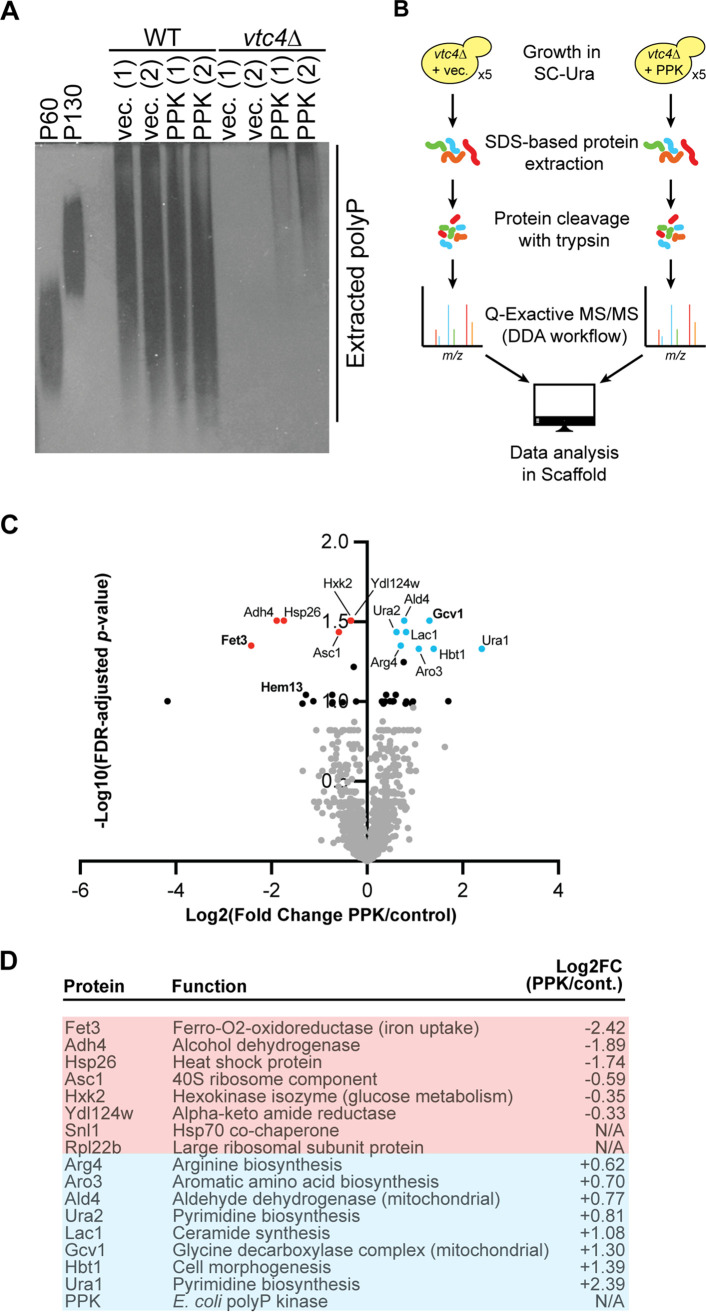
The proteome of PPK-expressing yeast. (A) PPK expression in yeast allows the production of polyP outside the vacuole. Polyphosphate was extracted from the strains expressing the indicated constructs and visualized using DAPI staining following separation on a 15.8% acrylamide–TBE–urea gel. Two biological replicates are presented for each condition. (B) Workflow for data-dependent acquisition (DDA)-based label-free mass spectrometry analysis. (C) Volcano plot with proteins up- or downregulated following PPK expression in a *vtc4*Δ background. Red proteins are downregulated (adjusted *P* value of <0.05), and blue proteins are upregulated (adjusted *P* value of <0.05). Black proteins are those with an adjusted *P* value of ≤0.1. (D) Table of the top differentially expressed proteins identified with an adjusted *P* value of <0.05. Images are representative of data from ≥3 experiments. FC, fold change; N/A, not applicable.

To determine why PPK expression and the accumulation of nonvacuolar polyP are toxic in yeast, we used label-free mass spectrometry to survey changes to the proteome in strains expressing PPK versus the empty vector control. Our initial experiments were carried out in a *vtc4*Δ background, which enabled us to confirm the production of the polyP in each isolate used for analysis, as judged by the lysine polyphosphorylation of the Fpr3 protein (15) (see Fig. S1A in the supplemental material). The polyP made by PPK in yeast was of the long-chain type, like that produced by E. coli under starvation (Fig. 1A) (16). Total protein from 5 isolates of the control and 5 isolates of PPK-expressing cells was isolated using an SDS lysis protocol prior to trypsinization and tandem mass spectrometry (MS/MS) analysis using a Thermo Scientific Q Exactive Plus Orbitrap mass spectrometer (Fig. 1B). As expected, peptides from bacterial PPK were detected only in the PPK-expressing strains (Table S1B). At the level of a false discovery rate (FDR)-adjusted *P* value of <0.05, 8 proteins were deemed significantly upregulated, while 6 were downregulated (Fig. 1C and D). An additional 24 proteins showed up- or downregulation with an associated statistical significance of 0.05 ≤ *P ≤ *0.1 (Fig. 1C and Table S1A). Finally, there were 2 proteins (Snl1 and Rpl22b) that were detected in all five replicates of the empty vector control but not in any replicates of the PPK-expressing strains (Fig. 1D and Table S1B).

10.1128/mbio.00390-22.1 FIG S1 Changes to the S. cerevisiae proteome with ectopic PPK expression. (A) PPK expression restores polyphosphate levels in samples used for mass spectrometry. Protein extracts were prepared from the indicated strains carrying either control or PPK-expressing plasmids using an SDS lysis protocol and separated on a NuPAGE gel. Separated proteins were transferred to a PVDF membrane before probing with an antibody that recognizes yeast Fpr3. Ponceau S staining of the membrane is shown as a loading control. The shift observed in PPK-expressing samples is a sign of lysine polyphosphorylation and polyP production. (B) Supplemental protein expression validation of mass spectrometry candidates in a *vtc4*Δ background. (C) Supplemental protein expression validation of mass spectrometry candidates in a wild-type background. (D) Supplemental protein expression validation of mass spectrometry candidates in a *vtc4*Δ background with mutant PPK included. Asterisks indicate nonspecific bands. (E) Expression levels of HA-PPK and mutant HA-PPK expressed from plasmids in a *vtc4*Δ background. These cell extracts were prepared from Fet3-3FLAG-tagged strains. (F) Supplemental protein expression validations of mass spectrometry candidates using the Gal-inducible HA-PPK system. All protein extracts from the indicated strains in panels B to F were prepared using a TCA lysis protocol and separated on SDS-PAGE gels. Separated proteins were transferred to a PVDF membrane before probing with an anti-FLAG antibody. Ponceau S staining of the membrane is used as a loading control. Images are representative of results from ≥2 experiments. Download FIG S1, file, MB. Copyright © 2022 McCarthy et al. 2022 McCarthy et al. https://creativecommons.org/licenses/by/4.0/ This content is distributed under the terms of the Creative Commons Attribution 4.0 International license.

10.1128/mbio.00390-22.6 TABLE S1 Mass spectrometry data. Download Table S1, XLSX file, 2.2 MB. Copyright © 2022 McCarthy et al. 2022 McCarthy et al. https://creativecommons.org/licenses/by/4.0/ This content is distributed under the terms of the Creative Commons Attribution 4.0 International license.

### Vacuolar polyP does not impact the cellular response to PPK expression.

**FIG 2:**
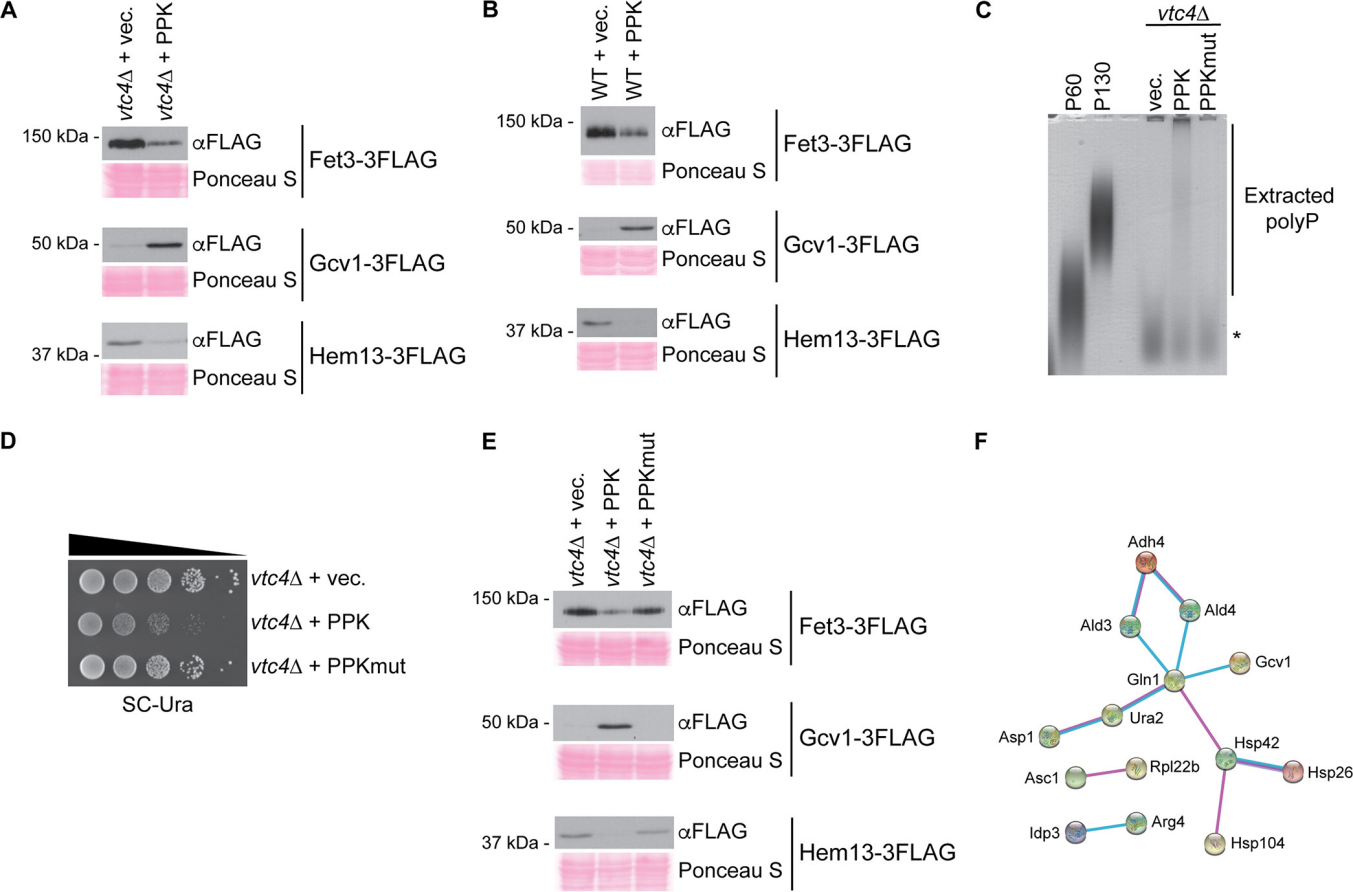
The response to PPK expression is not impacted by the presence of vacuolar polyP. (A and B) Protein extracts from the indicated strains were prepared using a TCA lysis protocol and separated on SDS-PAGE gels. Separated proteins were transferred to a PVDF membrane prior to probing with anti-FLAG antibody to detect candidate proteins. Ponceau S staining of the PVDF membrane is shown as a loading control. (C) Polyphosphate was extracted from the strains expressing the indicated constructs and visualized using toluidine blue staining following separation on a 15.8% acrylamide–TBE–urea gel. The asterisk indicates nonspecific staining of an unknown product that is not polyP, as it is present in *vtc4*Δ cells. (D) Tenfold dilutions of the indicated strains were spotted onto SC-Ura medium and incubated for 48 h at 30°C. (E) Protein extracts from the indicated strains were analyzed as described above for panel A. (F) STRING analysis locates differentially expressed proteins in subnetworks of physically interacting proteins. Magenta, experimental evidence; cyan, database evidence; purple, database evidence (specifically protein homology). Images are representative of results from ≥3 experiments.

To validate our proteomics data set, we expressed up- or downregulated proteins as C-terminal 3FLAG fusions from their endogenous loci and transformed these strains with control or PPK-expressing vectors. We first carried out this analysis in a *vtc4*Δ background, mirroring the conditions used for our mass spectrometry experiments. For differentially expressed proteins with a *P* value of *≤*0.1, we confirmed 6 of 6 upregulations chosen for analysis (e.g., Gcv1) and 3 of 4 downregulations chosen for analysis (e.g., Fet3 and Hem13), for an overall confirmation rate of 90% (Fig. 2A, Table S2, and Fig. S1B and D). The validation of protein changes in the ranges of both a *P* value of <0.05 and a *P* value of *≤*0.1 supports our use of the cutoff of a *P* value of *≤*0.1 for downstream bioinformatics analyses (see below). Notably, we also confirmed 5 of 6 differentially expressed proteins beyond the range of a *P* value of 0.1 (Table S2 and Fig. S1B and D), highlighting the overall high quality of our data set and the stringency of our statistical approach.

10.1128/mbio.00390-22.7 TABLE S2 Summary of MS data set confirmations. Download Table S2, XLSX file, 0.01 MB. Copyright © 2022 McCarthy et al. 2022 McCarthy et al. https://creativecommons.org/licenses/by/4.0/ This content is distributed under the terms of the Creative Commons Attribution 4.0 International license.

To test if these changes are influenced by the presence of vacuolar polyP, we repeated our Western blot analyses in a wild-type strain (i.e., *VTC4*^+^), with a confirmation rate of 86% (*P ≤ *0.1) (Fig. 2B, Table S2, and Fig. S1C). This observation is consistent with our previous work demonstrating that PPK expression sensitizes cells to rapamycin and cycloheximide regardless of *VTC4* status (15). As suggested previously (5), PPK is not simply replacing polyP that is missing in *vtc4*Δ cells. Instead, nonvacuolar polyP appears to have unique consequences for the cell. Finally, to test if the catalytic activity of PPK was required for the observed effects, we generated a mutant version of the enzyme where three histidine residues shown or proposed to be required for catalytic activity (17–19) were mutated to alanine. This mutant enzyme did not produce detectable polyP *in vivo* (Fig. 2C). Furthermore, it did not result in the same slow-growth defects seen for wild-type PPK expression (Fig. 2D). For 8 of 8 candidates tested (*P ≤ *0.1), mutant PPK did not impact protein levels (Fig. 2E, Table S2, and Fig. S1D) despite being expressed at higher levels than wild-type PPK (Fig. S1E). One interesting exception (although beyond the cutoff of a *P* value of *≤*0.1) is Ctt1-3FLAG, which was also upregulated in strains expressing the catalytic mutant (Table S2 and Fig. S1D). Still, our data confirm that PPK catalytic activity seems to be required for most changes measured. Finally, we note that proteins encoded by the phosphate (PHO) regulon (e.g., Pho8, Pho86, and Vtc2) (20) did not show significant changes in PPK-expressing cells (Table S1A). Therefore, the effects of PPK expression are not easily explained by changes in phosphate signaling.

### Expression changes for proteins involved in fundamental metabolism in PPK-expressing cells.

To identify the molecular functions impacted by PPK expression, we carried out Gene Ontology (GO) (21) enrichment analysis of up- or downregulated proteins (*P ≤ *0.1). We found a statistically significant enrichment (FDR-adjusted *P* value of 0.036) of GO:0006082 (organic acid metabolic process) (Table S1F). Differentially expressed proteins belonging to this group include those involved in glucose metabolism (Hxk2 and Tdh3), B vitamin synthesis (Snz1 and Fol2), and amino acid biogenesis or turnover (e.g., Aro3, Gcv1, and Gln1) (22). These changes suggest that polyP accumulation outside the vacuole influences important aspects of primary metabolism. Using STRING (Search Tool for the Retrieval of Interacting Genes/Proteins) (23) to model protein interactions, we found that proteins involved in amino acid metabolism had physical interactions with each other and with 3 heat shock proteins (Hsp26, Hsp42, and Hsp104) (22) that were also significantly up- or downregulated with PPK expression (Fig. 2F). The identification of heat shock proteins in this network is interesting in light of the proposed role of polyP itself as a molecular chaperone (24). Other heat shock or “quality control” proteins up- or downregulated in our data set include Hsp12, Hsp104, and Snl1, a cochaperone for Hsp70 (22).

PolyP can function as an ion chelator, and recent work from Beaufay et al. suggests that it can act as an inhibitor of the Fenton reaction, in which Fe^2+^ reacts with hydrogen peroxide to form reactive hydroxyl radicals (25). Interestingly, we noted that a number of proteins downregulated with PPK expression either are involved in ion transport (e.g., Fet3, which oxidizes Fe^2+^ to Fe^3+^ [26]) or require divalent ions as cofactors (e.g., Adh4, which requires zinc for its catalytic activity [27]). Genes encoding other differentially expressed proteins confer resistance to metal ions (e.g., *HIS1* [28]) or are induced in response to metal deficiency (e.g., *ADH4*, induced in response to low zinc [29]). While the toxicity associated with PPK expression was not impacted by the addition of various divalent cations (Fe^2+^, Mg^2+^, Zn^2+^, and Ca^2+^) to growth media (Fig. S2), we cannot yet rule out that disruption of ion homeostasis impacts proteome changes independently of the overt toxicity observed in PPK-expressing cells.

10.1128/mbio.00390-22.2 FIG S2 Supplementing media with excess metals does not rescue PPK-induced growth defects. The indicated strains were spotted in 10-fold dilution series. All metals were used at a concentration of 200 μM with the exception of calcium chloride, which was used at 100 mM. Plates were incubated at 30°C for 48 h. Images are representative of results from 2 experiments, except for calcium chloride, which is representative of results from 3 experiments. Download FIG S2, TIF file, 1.7 MB. Copyright © 2022 McCarthy et al. 2022 McCarthy et al. https://creativecommons.org/licenses/by/4.0/ This content is distributed under the terms of the Creative Commons Attribution 4.0 International license.

### An Msn2/Msn4-dependent signaling pathway limits viability in PPK-expressing cells.

**FIG 3:**
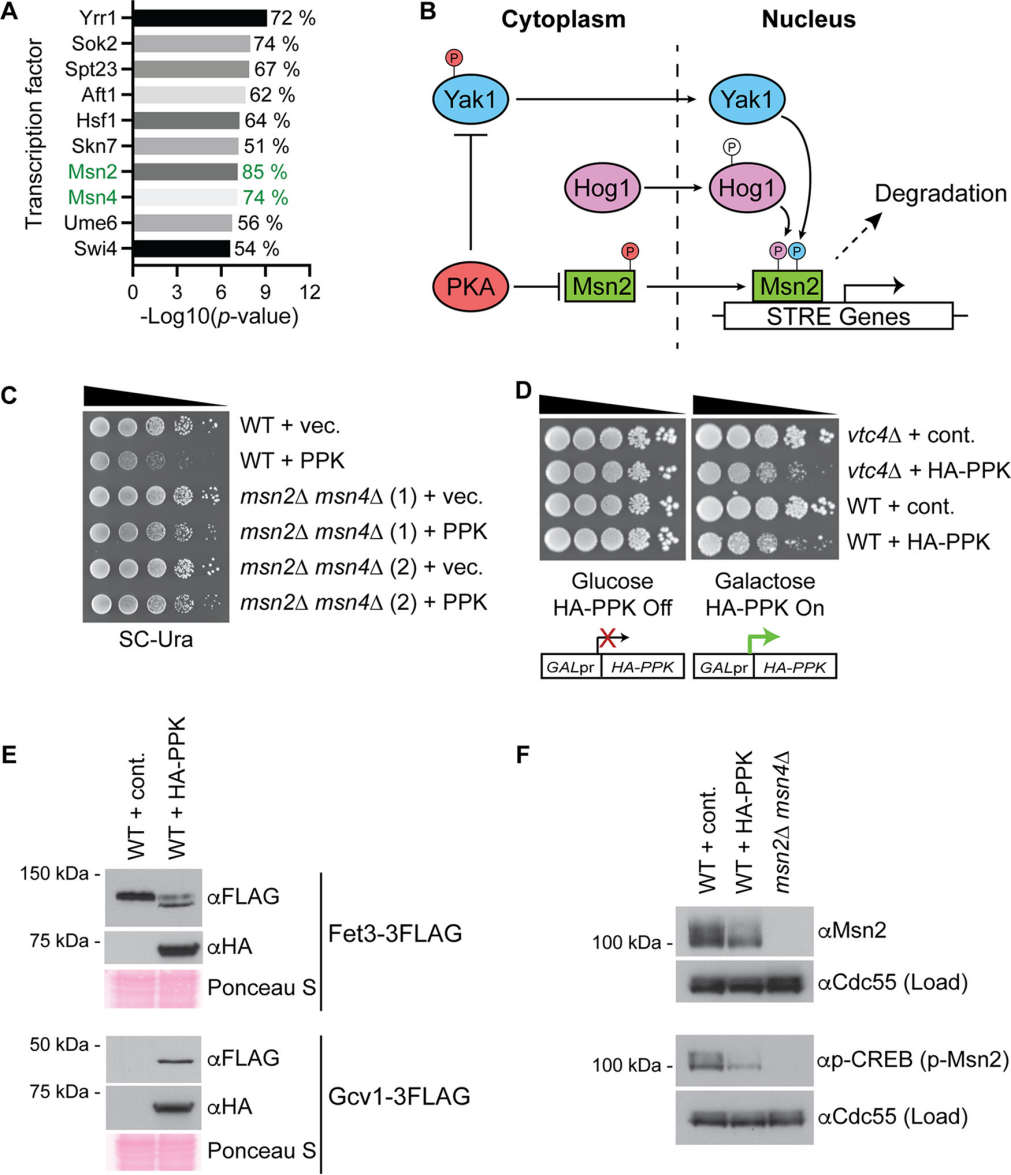
The Msn2 and Msn4 transcription factors limit viability in PPK-expressing cells. (A) YEASTRACT analysis was used to identify candidate transcriptional regulators based on proteins up- and downregulated in the mass spectrometry analysis. See Materials and Methods for details. The percentages show the fractions of differentially expressed proteins (adjusted *P* value of ≤0.1) whose encoding genes are regulated by the indicated transcription factors. (B) Relationship between PKA/Yak1/Hog1 and Msn2/Msn4 in stress signaling. (C) Tenfold serial dilutions of the indicated strains transformed with an empty or PPK-expressing vector were spotted onto the indicated media and incubated for 48 h at 30°C. Two biological replicates for the *msn2*Δ *msn4*Δ mutant are shown for each condition. (D) Schematic of an inducible HA-PPK expression system under the control of the GAL promoter. Gal-induced HA-PPK expression results in decreased growth in both the *vtc4*Δ and wild-type backgrounds. Tenfold serial dilutions of the indicated strains were spotted onto the indicated media and incubated for 72 h at 30°C. (E) Protein extracts from the indicated strains harboring an empty GAL promoter or the inducible HA-PPK expression system were prepared using a TCA lysis protocol and separated on SDS-PAGE gels. Separated proteins were transferred to a PVDF membrane prior to the detection of proteins with the indicated antibodies. Ponceau S staining of the PVDF membranes is shown as a loading control. (F) Protein extracts from the indicated strains were prepared and analyzed as described above for panel E. A phospho-CREB antibody was used to detect the NLS-specific phosphorylation of Msn2. Cdc55 is used as a loading control. Images are representative of results from ≥3 experiments.

We next used the Yeast Search for Transcriptional Regulators and Consensus Tracking (YEASTRACT) (30) tool to identify candidate transcription factors involved in the regulation of genes encoding proteins up- or downregulated in our data set (Fig. 3A). We were particularly interested in Msn2 (regulating 85% of hits) and its paralog Msn4 (regulating 74% of hits) for two reasons. First, these transcription factors are involved in the response to diverse cellular stresses via the activation of stress response element (STRE) genes (31, 32) (Fig. 3B). Second, the constitutive activation of Msn2 is associated with slow growth (33, 34). We reasoned that the misregulation of Msn2 and/or Msn4 impacts cell growth and/or division in PPK-expressing cells. Indeed, the deletion of *MSN2* and *MSN4* in PPK-expressing strains improved growth in serial dilution assays (Fig. 3C), suggesting that these transcription factors hinder growth in the presence of nonvacuolar polyP. We next examined changes to Msn2 via Western blotting. For these experiments, we developed an integrated galactose (Gal)-inducible version of the PPK expression system (Fig. 3D). This allowed better control over PPK expression and prevented strains from adapting to the presence of nonvacuolar polyP. The PPK construct contains an N-terminal hemagglutinin (HA) epitope tag, which allowed us to monitor expression in mutant strains. Notably, Gal-induced HA-PPK expression resulted in growth defects in both the *vtc4*Δ and WT strain backgrounds (Fig. 3D). HA-PPK-synthesized polyP was quantified in *vtc4*Δ cells and amounted to approximately 50% of that produced in wild-type cells grown in galactose (Fig. S3A). We further validated our Gal-inducible system by demonstrating that Gcv1-3FLAG and Hsp42-3FLAG are upregulated and that Fet3-3FLAG and Adh4-3FLAG are downregulated following HA-PPK expression (Fig. 3E and Fig. S1F), mirroring what was observed for the plasmid-based expression system used in previous experiments. The loss of PKA-dependent Msn2 nuclear localization signal (NLS) phosphorylation is associated with its nuclear localization and transcriptional activation of stress response element genes (35) (Fig. 3B). HA-PPK-expressing cells had lower levels of this phosphorylated form of Msn2, which can be fortuitously detected using an antibody against human pCREB (35, 36) (Fig. 3F). Notably, the levels of total Msn2 were also reduced with PPK expression (Fig. 3F), an observation that may seem at odds with the idea of enhanced Msn2 activity. However, we note that nuclear Msn2 is thought to be destabilized under conditions of chronic stress and low PKA activity as well as heat shock (34, 37). Importantly, Msn2/Msn4-induced genes whose encoded proteins were upregulated with PPK expression showed increased mRNA expression, consistent with regulation occurring at the transcriptional level (Fig. S3B).

10.1128/mbio.00390-22.3 FIG S3 PolyP quantification, qPCR analysis of Msn2/Msn4 targets, and HA-PPK levels in the *HOG1* and *YAK1* mutants. (A) Quantification of polyP produced by the Gal-inducible HA-PPK system. Extracted polyP was quantified from 5 biological replicates using the Phosfinity kit. Error bars represent standard deviations. Significance was determined by 2-tailed Student’s *t* test. (B) qPCR analysis of differentially expressed proteins regulated by the Msn2/Msn4 transcription factors. qPCR was performed on 5 biological replicates. Error bars represent standard errors of the means. The indicated *P* values were determined by Bio-Rad CFX Maestro 2.3 version 5.3.022.1030 software, which uses Student’s *t* test based on the geometric mean. (C) Protein extracts from the indicated strains were prepared using a TCA lysis protocol and separated on SDS-PAGE gels. Separated proteins were transferred to a PVDF membrane and probed with anti-HA to detect HA-PPK. Ponceaus S staining is shown as a loading control. (D) The indicated strains were spotted in 10-fold dilution series, and plates were incubated at 30°C for 48 h. (E) Protein extracts from the indicated strains were prepared using a TCA lysis protocol and separated on SDS-PAGE gels. Separated proteins were transferred to a PVDF membrane and probed with anti-HA to detect HA-PPK. Ponceaus S staining is shown as a loading control. (F) PolyP levels are unaffected in *hog1*Δ and *yak1*Δ mutants. Polyphosphate was extracted from the indicated strains and visualized using toluidine blue staining following separation on a 15.8% acrylamide–TBE–urea gel. The asterisk indicates nonspecific staining of an unknown product that is not polyP, as it is present in *vtc4*Δ cells. Images are representative of results from ≥3 experiments, except for panel E, which is representative of results from 2 experiments. Download FIG S3, TIF file, 2.2 MB. Copyright © 2022 McCarthy et al. 2022 McCarthy et al. https://creativecommons.org/licenses/by/4.0/ This content is distributed under the terms of the Creative Commons Attribution 4.0 International license.

**FIG 4:**
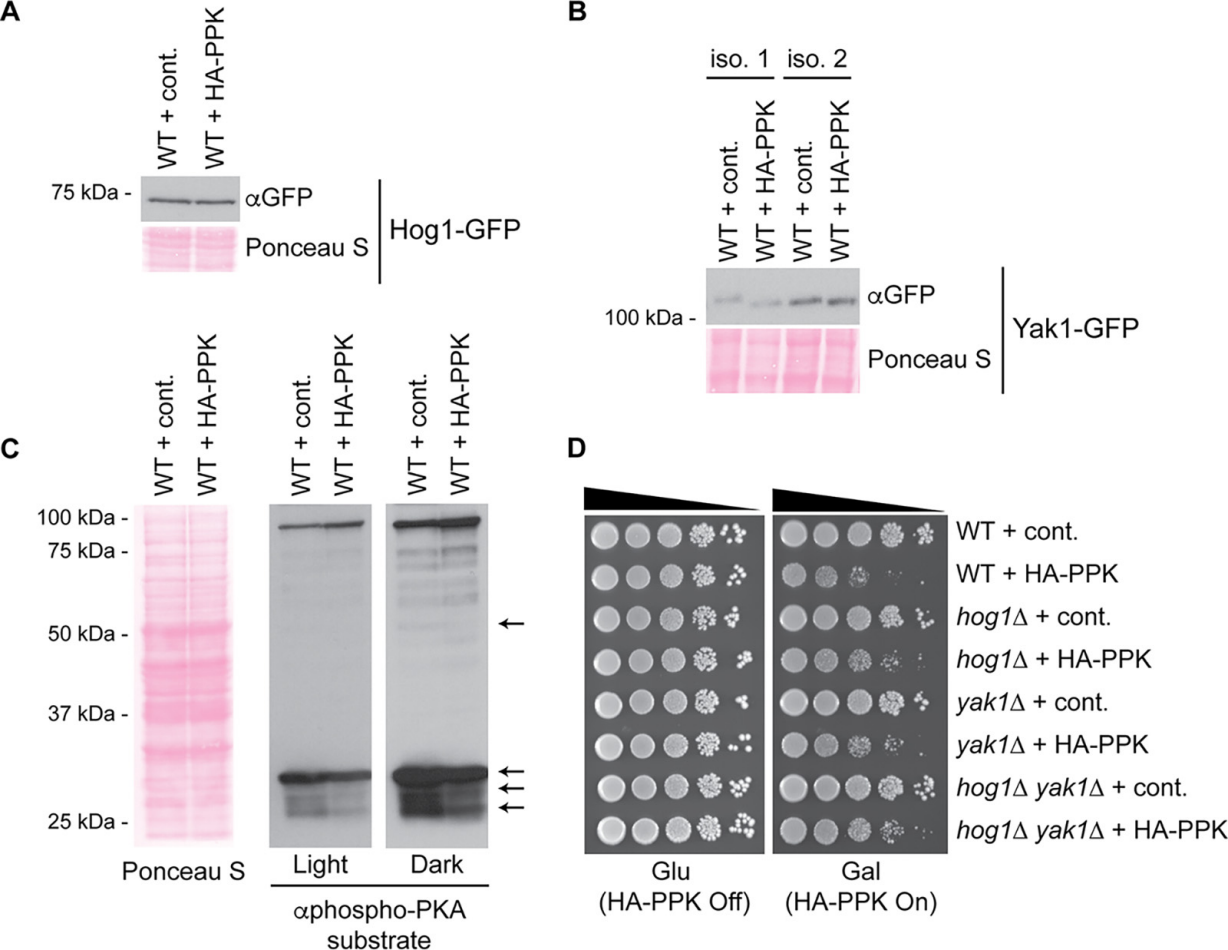
Yak1 and Hog1 kinases modulate the response to PPK expression. (A) Hog1-GFP levels are unaffected by PPK expression. Protein extracts from the indicated strains were prepared using a TCA lysis protocol and separated on SDS-PAGE gels. Separated proteins were transferred to a PVDF membrane before probing with the indicated antibodies to detect the proteins of interest. Ponceau S staining of the PVDF membrane is shown as a loading control. (B) PPK expression leads to an increase in the electromobility of Yak1. Protein extracts from the indicated strains were prepared and analyzed as described above for panel A, but proteins were run on a 10% SDS-PAGE gel with a 77:1 acrylamide-to-bis ratio. Two biological replicates are shown for each condition. (C) Phosphorylated PKA substrates were detected using an antibody that detects phosphorylated serine or threonine residues with arginine at the −3 and −2 positions (RRXS*/T*). Arrows indicate misregulated phosphorylated PKA substrates. Ponceau S staining of the PVDF membrane is shown as a loading control. (D) Tenfold serial dilutions of the indicated strains were spotted onto the indicated media and incubated for 72 h at 30°C. Images are representative of results from ≥3 experiments.

Hog1 and Yak1 are upstream kinases that act on Msn2 to regulate its nuclear import and at the promoter level to stimulate stress-dependent gene induction (38, 39) (Fig. 3B). Therefore, we next tested if Hog1 and Yak1 might be involved in the response to HA-PPK expression and polyP accumulation outside the vacuole. While we saw no change in the expression of Hog1-green fluorescent protein (GFP) or Yak1-GFP (Fig. 4A and B), the electrophoretic mobility of Yak1-GFP was slightly but reproducibly increased with PPK expression (Fig. 4B). We surmised that this could represent a loss of autophosphorylation or PKA-dependent phosphorylation that accompanies Yak1 activation or translocation to the nucleus, respectively (40). Indeed, some PKA-dependent phosphoproteins show misregulation in PPK-expressing cells (Fig. 4C). The deletion of *HOG1* or *YAK1* partially rescued the slow growth associated with PPK expression (Fig. 4D). As a control, these strains had equal levels of HA-PPK expression (Fig. S3C). We also observed a comparable effect on cells deleted for *VTC4*, which were confirmed to accumulate similar levels of PPK-synthesized polyP (Fig. S3D to F). Collectively, our data suggest that Hog1 and Yak1 may signal through Msn2/Msn4 in response to polyP accumulation outside the vacuole and that the activation of this signaling pathway inhibits growth. However, we cannot completely rule out a role for these proteins in the metabolism or distribution of polyP itself.

### Ddp1 cooperates with Ppx1 to degrade nonvacuolar polyP.

**FIG 5:**
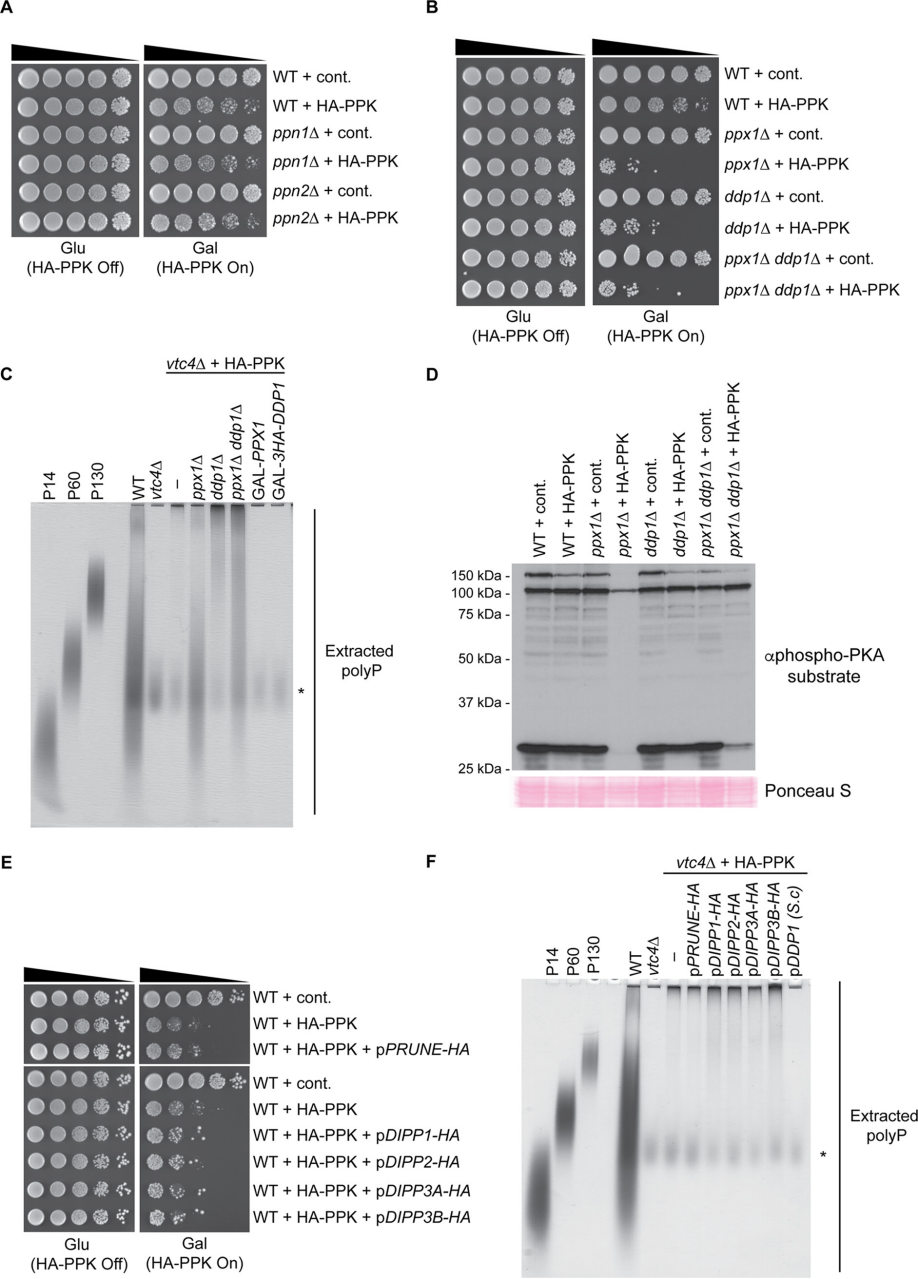
PolyP accumulation outside the vacuole is countered by Ppx1 and Ddp1. (A and B) The indicated strains were spotted in a 5-fold dilution series onto plates containing 2% glucose or 1.8% raffinose with 0.2% galactose and incubated at 30°C for 72 h. (C) Polyphosphate was extracted from the indicated strains and visualized using toluidine blue staining following separation on a 15.8% acrylamide–TBE–urea gel. The asterisk indicates nonspecific staining of an unknown product that is not polyP, as it is present in *vtc4*Δ cells. (D) PKA activity is drastically reduced in *ppx1*Δ cells expressing PPK. Protein extracts from the indicated strains were prepared using a TCA lysis protocol and separated on SDS-PAGE gels. Separated proteins were transferred to a PVDF membrane before probing with a phospho-PKA substrate antibody. Ponceau S staining of the membrane is used as a loading control. In these experiments, protein levels seem to be lower in PPK-expressing strains, which may be indicative of cell death. (E) Strains with the indicated plasmids expressing yeast or candidate human polyphosphatase enzymes were spotted in 10-fold dilution series and incubated at 30°C for 72 h. (F) Polyphosphate was extracted from the strains expressing the indicated plasmids and analyzed as described above for panel D. Images are representative of results from ≥3 experiments.

Since aberrant polyP outside the vacuole leads to changes in the expression of the proteome, we expect that mechanisms may exist to counter its accumulation. In yeast, work on polyphosphatase enzymes has focused on four proteins: Ppn1, Ppn2, Ppx1, and Ddp1. Ppn2 and Ddp1 are thought to act as endopolyphosphatases, which cleave polyP chains internally (2). Ppx1 functions as an exopolyphosphatase that cleaves polyP chains starting at the end (2). Ppn1 has been proposed to have both functions (2). Work by Gerasimaitė et al. demonstrated that *GAL*pr-*PPX1* strains show decreased growth when grown in glucose (repressed *PPX1*) versus galactose (*PPX1* overexpression) in the presence of nonvacuolar polyP (5). Whether Ppx1 works alone or in conjunction with additional polyphosphatases is unknown. Therefore, we tested the contribution of each of the four polyphosphatase enzymes in our system. The deletion of the polyphosphatase gene *PPN1* or *PPN2* had no impact on the growth or viability of HA-PPK-expressing cells (Fig. 5A). This observation is consistent with their roles as vacuolar enzymes and the lack of an impact of vacuolar polyP stores on PPK-induced toxicity (Fig. 3D). In contrast, and as expected, *PPX1* deletion greatly enhanced the toxicity associated with PPK expression (Fig. 5B). Interestingly, a similar phenotype was observed in cells disrupted for *DDP1*. In contrast to the slow-growth phenotype conferred by PPK expression alone, the deletion of these genes resulted in a dramatic decrease in viability in addition to slow growth (Fig. 5B). Interestingly, the simultaneous deletion of *DDP1* and *PPX1* showed an effect that was not more dramatic than that observed for either single mutant (Fig. 5B). This largely epistatic relationship was recapitulated in the context of a liquid growth assay (Fig. S4A), suggesting that Ddp1 and Ppx1 function in the same pathway to counter the toxicity of polyP outside the vacuole. However, we note that HA-PPK expression levels are lower in the *ppx1*Δ *ddp1*Δ double mutant than in wild-type cells or either single mutant (Fig. S4C and G), which somewhat complicates this interpretation. To test if Ddp1 and Ppx1 physically interact to degrade polyP in a concerted fashion, we attempted to coimmunoprecipitate 3HA-Ddp1 and Ppx1 but did not detect any interaction between the two proteins (Fig. S4B).

10.1128/mbio.00390-22.4 FIG S4 HA-PPK regulation in polyphosphatase mutants. (A) Growth curves for the indicated strains were generated using the Bioscreen C system described in Materials and Methods. (B) Ppx1 does not coimmunoprecipitate with Ddp1. 3HA-Ddp1 was immunoprecipitated using an anti-HA antibody and then recovered using protein A-agarose beads before elution in sample buffer. Eluted proteins from the indicated strains were separated on SDS-PAGE gels and transferred to a PVDF membrane prior to probing with anti-HA to detect 3HA-Ddp1 and anti-Ppx1 to detect Ppx1. (C) HA-PPK levels in polyphosphatase mutants. Protein extracts from the indicated strains were prepared using a TCA lysis protocol and separated on SDS-PAGE gels. Separated proteins were transferred to a PVDF membrane and probed with anti-HA to detect HA-PPK. Ponceau S staining is shown as a loading control. (D) The indicated strains were spotted in 10-fold dilution series, and plates were incubated at 30°C for 48 h. (E to G) After a 1-h galactose induction of HA-PPK, lysine polyphosphorylation of Fpr3 and Rts1 was analyzed by NuPAGE, followed by transfer to a PVDF membrane and the detection of proteins using the indicated antibodies. Ponceau S staining of the membrane is used as a loading control. Images are representative of results from ≥3 experiments for all panels except panel B, which is representative of results from 2 experiments. Download FIG S4, TIF file, 2.2 MB. Copyright © 2022 McCarthy et al. 2022 McCarthy et al. https://creativecommons.org/licenses/by/4.0/ This content is distributed under the terms of the Creative Commons Attribution 4.0 International license.

To understand the mechanisms of Ppx1 and Ddp1 action, we analyzed polyP from our panel of polyphosphatase mutants. These experiments were conducted in a *vtc4*Δ background to allow the visualization of HA-PPK-synthesized polyP rather than the VTC-synthesized polyP stored in the vacuole but released during the polyP isolation protocol. As we observed in the plasmid-based system, HA-PPK-synthesized polyP accumulated as predominantly long chains reminiscent of those synthesized by E. coli in response to cellular stress (Fig. 5C). Here again, the amount of polyP is smaller than that in a wild-type yeast cell, consistent with the notion that it is the localization of PPK-synthesized polyP rather than the amount of that polyP that drives changes in cell growth (5). HA-PPK-expressing strains lacking *PPX1* had more polyP, and the chains were of a medium length (Fig. 5C). Strains lacking *DDP1* also showed increased polyP, but these chains were of the long form (Fig. 5C). The *ppx1*Δ *ddp1*Δ double mutant accumulated both medium and long polyP chains, with long chains being predominant. The overexpression of either enzyme reduced the level of HA-PPK-synthesized polyP to background levels (Fig. 5C) and rescued the slow growth associated with PPK expression (Fig. S4D). While these experiments suggest that Ddp1 and Ppx1 act sequentially to degrade long chains of polyP, Ppx1 may also target a distinct population of medium-length chains synthesized by HA-PPK. Interestingly, PKA activity was drastically reduced in *ppx1*Δ cells expressing HA-PPK (Fig. 5D). This observation reinforces the connection between polyP accumulation outside the vacuole and signaling through this pathway. In contrast, the deletion of *DDP1* had little effect (Fig. 5D), suggesting that the impact on PKA may be chain length dependent.

To corroborate and extend our findings with yeast Ppx1 and Ddp1, we analyzed lysine polyphosphorylation of previously identified targets (15). Since lysine polyphosphorylation is a nonenzymatic modification, it is thought to be controlled largely by the concentration of polyP itself. We carried out these experiments in a *vtc4*Δ background to prevent the interference of vacuolar polyP, which can polyphosphorylate proteins during cell lysis (41). The deletion of *PPX1* or *DDP1* in HA-PPK-expressing cells resulted in the decreased electrophoretic mobility of the polyphosphorylation targets Rts1 and Fpr3 on NuPAGE gels, indicative of lysine polyphosphorylation (Fig. S4E to G) (15). Therefore, the “extra” polyP in these cells is competent to participate in lysine polyphosphorylation reactions. The decreased electrophoretic mobility observed in *ppx1*Δ and *ddp1*Δ cells could result from the modification of additional lysines at higher concentrations of polyP (42). The greater impact of *ddp1*Δ may reflect the addition of longer chains to individual target lysines in these cells. We propose that the enhanced toxicity of PPK expression observed in *ddp1*Δ and *ppx1*Δ mutants may be due in part to the aberrant polyphosphorylation of proteins in the cytoplasm, although the identities of these remain unknown.

Yeast Ppx1 and Ddp1 show homology to human Prune and DIPP1-DIPP3, respectively (43, 44). Prune and DIPP1-DIPP3 have exo- or endopolyphosphatase activity *in vitro* (43–45). Only DIPP1 (also called NUDT3) has been described to have activity *in vivo* (45). Samper-Martín et al. showed that DIPP1 overexpression confers a zinc-dependent reduction in polyP detected in HEK293T cells via a polyP binding probe (45). Conversely, DIPP1 knockdown caused an increase in polyP in both HEK293T and SH-SY5Y cells using this same method of detection (45). To determine if candidate polyphosphatases could impact PPK-induced toxicity and/or polyP accumulation in yeast, we expressed them in yeast under the control of the strong glyceraldehyde-3-phosphate dehydrogenase (GPD) promoter (Fig. S5A). In contrast to *DDP1* overexpression, the ectopic expression of human Prune or DIPP1-DIPP3 proteins did not improve growth in PPK-expressing cells (Fig. 5E) and did not decrease polyP accumulation (Fig. 5F). Since DIPP1 activity is thought to be zinc dependent (45), we added zinc to the growth media used for these experiments. Although the highest concentrations of zinc used caused a slight improvement in the growth of strains expressing HA-PPK alone or in the presence of Prune, the growth of DIPP1-DIPP3-expressing strains was unaffected (Fig. S5B). Similarly, although the addition of zinc resulted in a slight decrease in polyP levels in all strains tested, this was not impacted by the ectopic expression of DIPP1 (Fig. S5C). Either Prune and DIPP1-DIPP3 do not directly impact the accumulation of long-chain polyP *in vivo* or their activities are not faithfully recapitulated in yeast under the conditions tested.

10.1128/mbio.00390-22.5 FIG S5 Overexpression of potential mammalian polyphosphatases. (A) Protein extracts from the indicated strains were analyzed using a 12% SDS-PAGE gel following TCA-style lysis, prior to transfer to a PVDF membrane and detection with anti-HA to detect HA-PPK. Strains were transformed with the empty vector or HA-tagged Prune-, HA-tagged DIPP1-, HA-tagged DIPP2-, HA-tagged DIPP3A-, HA-tagged DIPP3B-, or yeast Ddp1-expressing plasmids. Ponceau S staining is shown as a loading control. (B) Strains with the indicated plasmids expressing potential mammalian polyphosphatases or yeast Ddp1 were spotted in 10-fold serial dilutions onto the indicated media supplemented with or without zinc sulfate. (C) Polyphosphate was extracted from strains expressing the indicated plasmids and visualized using toluidine blue staining following separation on a 15.8% acrylamide–TBE–urea gel. The asterisk indicates nonspecific staining of an unknown product that is not polyP, as it is present in *vtc4*Δ cells. Images are representative of results from 3 experiments. Download FIG S5, TIF file, 2.1 MB. Copyright © 2022 McCarthy et al. 2022 McCarthy et al. https://creativecommons.org/licenses/by/4.0/ This content is distributed under the terms of the Creative Commons Attribution 4.0 International license.

## DISCUSSION

### Nonvacuolar polyP reprograms the yeast proteome.

There are conflicting data concerning the concentration of polyP that accumulates outside the vacuole in wild-type yeast, although it is generally accepted that the vacuole harbors substantially higher concentrations than other areas of the cell (2). The sequestration of polyphosphate in specific subcompartments is not unique to yeast. Human cells accumulate high concentrations of polyP in the nucleolus or in dense granules of platelets (9, 11). Trypanosomes accumulate high levels of polyP in acidocalcisomes (46) as well as in glycosomes and the nucleolus in smaller amounts (47). Even in many bacteria, polyP produced in response to stress is concentrated in granules (2). While the concentration of polyP in these areas is important for specific cellular functions (i.e., blood clotting in the case of dense granules [48]), it is less clear if polyP is actively excluded from (or limited within) some areas of the cell and why this might be important. The expression of bacterial PPK in yeast, coincident with the production of polyP outside the vacuole, was previously demonstrated to reduce growth and sensitize cells to rapamycin and cycloheximide (5, 15). Our new study provides a critical advance by (i) identifying molecular pathways that mediate the toxic effects of PPK expression and (ii) providing molecular insight into how exo- and endopolyphosphatases cooperate to counter the accumulation of nonvacuolar polyP in yeast.

We are not able to rule out that other enzymatic activities of the PPK enzyme (e.g., the phosphorylation of nucleotides) contribute to the proteomic changes measured in our mass spectrometry experiments. However, the fact that PPK’s impact is greatly exacerbated in strains lacking the Ddp1 and/or Ppx1 polyphosphatase suggests that downstream changes in growth and/or viability are related to changes in polyP metabolism. PolyP could influence the expression or stability of some proteins directly, perhaps by binding to transcription factors or proteins themselves. We expect that the investigation of such direct modes of action will provide clues to the molecular functions of polyP that are broadly applicable, even under endogenous scenarios. Protein changes following PPK expression could also be indirect, for example, as a downstream result of polyP-mediated ion chelation or the cell’s response to more direct activities. Importantly, we consider such indirect effects to be equally interesting as they provide valuable insights into how eukaryotic cells respond to mislocalized polyP and may aid in the identification of scenarios when this occurs in the absence of PPK expression.

### Ddp1 and Ppx1 limit the toxic effects of nonvacuolar polyP.

**FIG 6:**
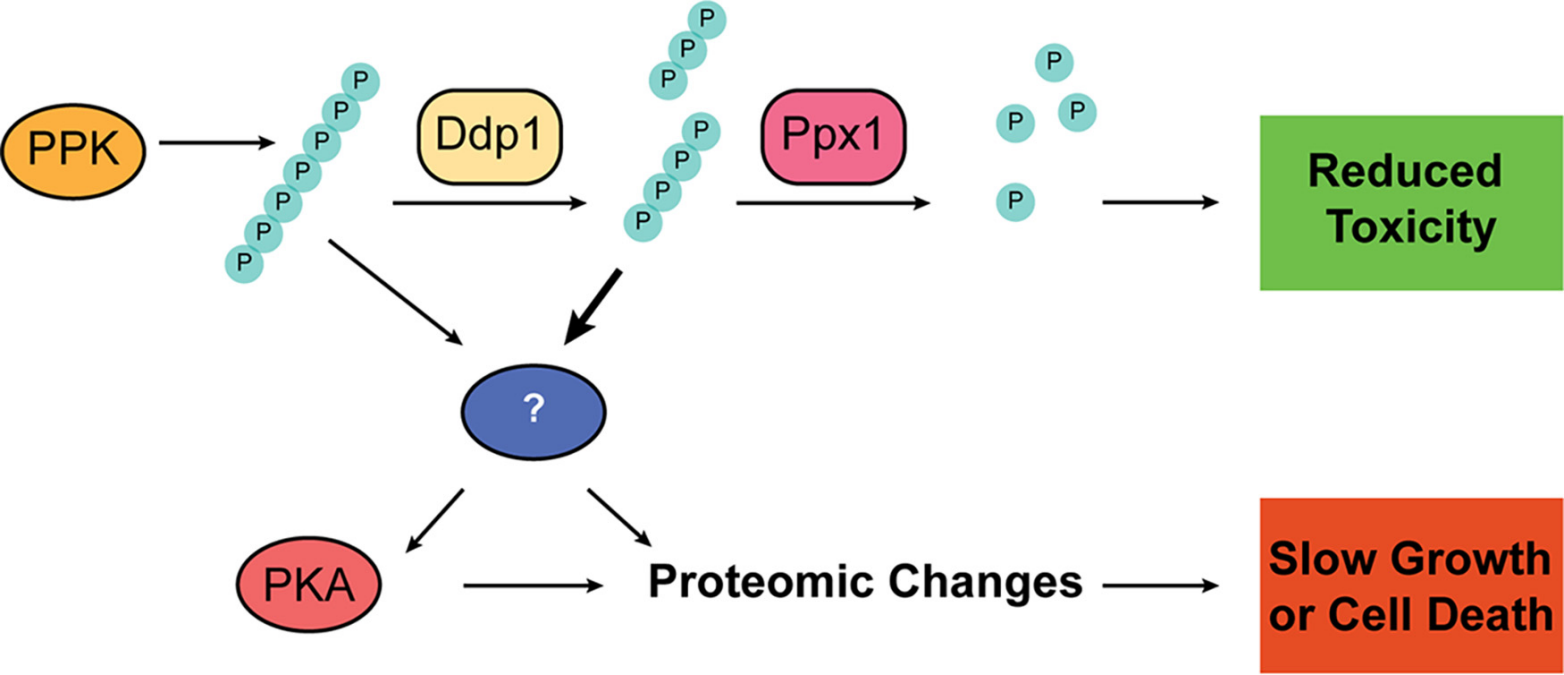
Working model for the cellular response to nonvacuolar polyP in yeast. PPK-produced polyP accumulates in the cytoplasm and slows growth by a variety of pathways, which include the activation of a Yak1/Hog1-Msn2/Msn4 signaling cascade. The accumulation of polyP outside the vacuole is countered by Ppx1 and Ddp1, which work together to degrade polyP. See the text for details.

In *ppx1*Δ and *ppx1*Δ *ddp1*Δ cells expressing PPK, polyP accumulates as medium-length chains, similar to those made by the VTC complex in wild-type cells. This argues against the idea that PPK expression is toxic only because it makes long chains that are not normally seen in yeast. However, we cannot rule out that different chain lengths have unique impacts on cell signaling and physiology, and this remains an important area for future research. Indeed, there is precedent for chain-length-dependent effects of polyP. For example, polyP chains of different lengths affect different parts of the blood coagulation cascade (49), and long chains preferentially stimulate changes in macrophage signaling important for the immune response (50). Since we have not recovered any evidence of a physical interaction between Ddp1 and Ppx1, we propose that they act sequentially to degrade long polyP chains (Fig. 6), with Ddp1 first cleaving chains to generate free polyP “ends” on which Ppx1 can then act. Notably, Ppx1 has also been shown to directly remove polyP chains from the nuclear polyphosphorylated target Nsr1 (51) and could act on other proteins in PPK-expressing cells to limit toxicity.

### PolyP as an activator of stress signaling.

We propose that polyP escaping degradation by polyphosphatases activates a stress-signaling pathway that limits cell growth or viability (Fig. 6). It is unclear if polyP is indeed causing some type of intracellular stress (i.e., the accumulation of unfolded proteins) or if it is inappropriately activating stress-signaling pathways in the absence of stress. Regardless, the downstream consequences appear to involve the PKA pathway, which plays a central role in the regulation of cell metabolism, growth, as well as diverse stress responses (52). Our results show that the accumulation of nonvacuolar polyP leads to the broad misregulation of PKA substrates. Some of these substrates may include differentially expressed proteins identified in our mass spectrometry experiments. For example, Ura2, which is downregulated in PPK-expressing cells, is phosphorylated by PKA *in vitro* (53). Moreover, PKA regulates the activity of Msn2/Msn4 and Yak1 through direct phosphorylation (35, 40), and recently, it was suggested that PKA might modulate the activation of the Hog1 pathway during impaired sphingolipid biosynthesis (54). Previous work has shown that the deletion of *YAK1* or *MSN2* and *MSN4* under conditions of low PKA activity rescues related slow-growth phenotypes (55, 56). We have shown that the deletion of *YAK1* or *MSN2* and *MSN4* also rescues growth in PPK-expressing cells, which is in accordance with scenarios of low PKA activity. Furthermore, low PKA activity imparted by the accumulation of nonvacuolar polyP could explain the sensitivity of PPK-expressing cells to rapamycin (15) since mutants with low PKA activity have increased sensitivity to this drug (57). Our results suggest that nonvacuolar polyP might affect PKA activity directly or indirectly and that these changes may explain the observed toxicity (Fig. 6).

### Endogenous functions of nonvacuolar polyP.

The polyphosphate functions outside the vacuole are poorly understood. The Ppx1 and Ppn1 polyphosphatases localize in part to the mitochondria, and here, polyP is suggested to play a role in energy homeostasis and metabolism (58). Within the nucleus, polyphosphorylation may also inhibit the interaction of the Top1 and Nsr1 proteins in addition to modulating their localization to the nucleolus (51). It is also tempting to speculate that the nuclear localization of polyphosphate underlies its poorly understood role in cell cycle regulation (59). How polyP that functions in or transits through the cytoplasm in a wild-type cell avoids degradation by polyphosphatases is an intriguing question. It may be that these polyP molecules are bound by proteins or small molecules that prevent enzyme access to the phosphoanhydride bonds. It is also possible that Ddp1 and Ppx1 become activated to degrade cytoplasmic polyP only when the concentration exceeds a particular threshold. We further speculate that polyP could be released from vacuoles following damage with lysosomotropic agents such as *N*-dodecylimidazole (60) or during sporulation, where the vacuole undergoes programmed lysis (61). Ddp1 and Ppx1 are ideally placed to regulate these responses.

### Regulation of polyP in human cells.

Our study suggests that the degradation of long-chain polyP in human cells will require the combined action of both endo- and exopolyphosphatases, akin to what we observed for Ddp1 and Ppx1. Mammalian polyphosphatases may also play a role in degrading long-chain polyP made by pathogenic bacteria, which has been suggested to enter cells during infection to reprogram macrophages and thereby subvert host defense mechanisms (50). The identities of intracellular human polyphosphatases had long remained unknown, but Prune and DIPP1-DIPP3 have emerged as candidates based on their *in vitro* activities (43, 44, 62). Notably, mutations that reduce Prune activity *in vitro* give rise to a rare disease called neurodevelopmental disorder with microcephaly, hypotonia, and variable brain anomalies (NMIHBA) (62). However, expressing either Prune or DIPP1-DIPP3 in yeast resulted in no appreciable change in viability and no obvious decrease in PPK-synthesized polyP. We note that the *in vitro* exopolyphosphatase activity of Prune is mostly against very short chains (3 to 4 phosphate units in length) (44, 62). In our experiments, Prune was also expressed at substantially lower levels than the DIPP1-DIPP3 proteins in yeast despite the fact that both constructs used the GPD promoter. Together, these observations could account for the lack of an effect observed in our system. More surprising is the observation that the expression of DIPP1 had no effect in either the presence or absence of zinc. This finding is seemingly at odds with the exciting work of Samper-Martín et al., who describe a zinc-dependent activity of DIPP1 (also called NUDT3) in HEK293T and SH-SY5Y cells (45). One exciting possibility is that DIPP1 or other mammalian polyphosphatase enzymes require additional cofactors besides zinc that are not normally present in yeast. Such proteins could serve as important regulators of *in vivo* polyphosphatase activity and should be a focus of future work.

## MATERIALS AND METHODS

### Yeast strains and handling.

Yeast strains were constructed using standard methods as previously described (63–65). The locations of gene deletion and tagging cassettes were confirmed using PCR analyses (65). The full genotypes of all yeast strains used in this work are listed in Table S3 in the supplemental material. Cells were grown in yeast extract-peptone-dextrose (YPD), YEP-raffinose, YEP-galactose, or synthetic complete (SC) medium as described below for individual experiments. In general, SC-Ura and SC-Leu media were used to maintain *URA3*-based and *LEU2*-based selection plasmids, respectively.

10.1128/mbio.00390-22.8 TABLE S3 Yeast strains used in this work. Download Table S3, XLSX file, 0.01 MB. Copyright © 2022 McCarthy et al. 2022 McCarthy et al. https://creativecommons.org/licenses/by/4.0/ This content is distributed under the terms of the Creative Commons Attribution 4.0 International license.

### Plasmids.

All plasmids used for this work are listed in Table S5A. All plasmids generated for this work are available from Addgene (www.Addgene.org).

10.1128/mbio.00390-22.10 TABLE S5 Plasmids and qPCR primers used in this work. Download Table S5, XLSX file, 0.01 MB. Copyright © 2022 McCarthy et al. 2022 McCarthy et al. https://creativecommons.org/licenses/by/4.0/ This content is distributed under the terms of the Creative Commons Attribution 4.0 International license.

### Polyphosphate extractions. (i) Cell growth.

Cells expressing the plasmids indicated in Fig. 1A and Fig. 2C were diluted to an optical density at 600 nm (OD_600_) of 0.2 in SC-Ura medium with 2% glucose and grown at 30°C with shaking until the OD_600_ reading reached a final value of 0.8 to 1. Cells in Fig. 5C and Fig. S3F were diluted to an OD_600_ of 0.2 in YEP medium with 2% raffinose and grown for 3 h before the addition of 2% galactose and growth for another 3 h. Cells expressing the plasmids indicated in Fig. 5F and Fig. S5C were diluted to an OD_600_ of 0.2 in SC-Ura medium with 2% raffinose and grown for 3 h before the addition of 2% galactose and growth for another 3 h.

### (ii) PolyP extraction.

The polyP isolation protocol was adapted from a protocol first described by Bru et al. (66) and described elsewhere (16). The protocol is repeated here using similar language. Yeast cell pellets of approximately 8 OD_600_ units in size were resuspended in 400 μL of cold LETS buffer (100 mM LiCl, 10 mM EDTA, 10 mM Tris-HCl [pH 7.4], 0.2% SDS), transferred to a screw-cap tube containing 600 μL of phenol (pH 8) and 150 μL of MilliQ water (mqH_2_O), and vortexed for 20 s. Samples were then heated for 5 min at 65°C and cooled on ice for 1 min. Once cooled, 600 μL of chloroform was added, and the samples were vortexed for 20 s. Vortexed samples were centrifuged at 13,000 × *g* for 2 min at room temperature, and the top layer was transferred to a new tube containing 600 μL of chloroform. Samples were vortexed for 20 s and centrifuged again at 13,000 × *g* for 2 min, and the top layer was transferred to a clean 1.5-mL tube. To degrade RNA and DNA, 2 μL of 10 mg/mL of RNase A and 2 μL of 10 mg/mL of DNase I were added to samples, and the samples were incubated at 37°C for 1 h. Digested samples were transferred to prechilled 1.5-mL tubes containing 1 mL of 100% ethanol and 40 μL of 3 M sodium acetate (pH 5.3), and polyP was left to precipitate at −20°C for at least 3 h or overnight. PolyP was pelleted by centrifugation at 13,000 × *g* for 20 min at 4°C, and the supernatant was discarded. The pellet was washed with 500 μL of cold 70% ethanol and centrifuged at 13,000 × *g* for an additional 5 min. The supernatant was discarded, and the pellets were air dried before being resuspended in 20 to 30 μL of mqH_2_O.

### (iii) Gel analysis.

Extracted polyP was diluted 1:1 with polyP loading buffer (10 mM Tris-HCl [pH 7], 1 mM EDTA, 30% glycerol, bromophenol blue), and 15 μL was loaded onto a 15.8% Tris-borate-EDTA (TBE)–urea gel (5.25 g urea, 7.9 mL 30% acrylamide, 3 mL 5× TBE, 150 μL 10% ammonium persulfate [APS], 15 μL *N*,*N*,*N*′,*N*′-tetramethylethylenediamine [TEMED]) and run at 100 V for 1 h 45 min in 1× TBE running buffer. For toluidine blue staining, the gel was incubated in fixative solution with toluidine blue (25% methanol, 5% glycerol, 0.05% toluidine blue) for 20 min and destained with fixative solution without toluidine blue. For 4′,6-diamidino-2-phenylindole (DAPI) staining, the protocol was adapted from the one described previously by Smith and Morrissey (67). The extracted polyP was diluted 1:60 for wild-type or 1:10 for *vtc4*Δ background strains with polyP loading buffer, and 15 μL was loaded. The gel was incubated in fixative solution with DAPI (25% methanol, 5% glycerol, 50 mM Tris base, 2 μg/mL DAPI) for 30 min. The gel was destained with fixative solution without DAPI for 1 h, changing solution once halfway through. The destained gel was exposed to 365 nM UV light on a transilluminator to photobleach the DAPI-polyP until it became dark. Pictures were taken with a Bio-Rad ChemiDoc UV transilluminator.

### Polyphosphate quantification.

Polyphosphate was quantified using the Phosfinity total polyphosphate quantification kit (Aminoverse) according to the manufacturer’s protocol.

### Label-free mass spectrometry.

The sample preparation and mass spectrometry protocols are based on an experimental setup that we have used previously for work in HEK293T cells (16). Similar language is used in the description here. Cells were diluted to an OD_600_ of 0.2 in SC-Ura medium with 2% glucose and grown at 30°C with shaking until an OD_600_ of 1 was reached. Yeast pellets of approximately 50 OD_600_ units in size were resuspended in 600 μL of lysis buffer (5% SDS and 50 mM triethylammonium bicarbonate [TEAB] supplemented with protease and phosphatase inhibitor tablets [Roche]), and a 2-mL screw-cap tube was filled with acid-washed glass beads. Cells were lysed with 12 1-min pulses on a BioSpec minibeadbeater with incubation on ice in between pulses. The lysates were clarified by centrifugation at 15,000 × *g* for 10 min at 4°C. The supernatant was collected and centrifuged again at 15,000 × *g* for 10 min at 4°C before being collected again and frozen at −80°C. Frozen protein extracts were shipped overnight on dry ice to the UC Davis Proteomics core. Trypsin digestion was performed using S-Trap Mini spin columns (Protifi) according to the manufacturer’s protocol.

### Liquid chromatography and mass spectrometry analysis.

At UC Davis, digested peptides were first separated using Proxeon Easy-nLC II high-performance liquid chromatography (HPLC) (Thermo Scientific) coupled to a Proxeon nanospray source. The peptides were loaded onto a 100-μm by 25-mm Magic C_18_ 100-Å 5-micron reverse-phase trap and desalted online prior to being separated using a 75-μm by 150-mm Magic C_18_ 200-Å 3-micron reverse-phase column. For all runs, peptides were eluted using a 140-min gradient with a flow rate of 300 nL/min into a Thermo Scientific Q Exactive Plus Orbitrap mass spectrometer. First, an MS survey scan was obtained for the *m/z* range of 350 to 1,600. MS/MS spectra were acquired with the top 15 ions in the MS spectra subjected to higher-energy collisional dissociation, with resolutions at 400 *m/z* of 70,000 and 17,500 at the MS1 and MS2 levels, respectively. For precursor ion selection, an isolation mass window of 1.6 *m/z* was used. A normalized collision energy of 27% was used for fragmentation. Dynamic exclusion was set for 15 s.

### Bioinformatics analyses.

For mass spectrometry data, the bioinformatics pipeline used for analysis is similar to the one previously described (16). The details are presented here with minimal rewording for clarity.

### Protein identification.

Tandem mass spectra were extracted using the msConvert program (68). X!Tandem (version X!Tandem ALANINE [2017.2.1.4]; thegmp.org) was used to analyze MS/MS spectra and identify peptide sequences (69). The X!Tandem program was used to search the Swiss-Prot/UniProt S. cerevisiae database (70) (proteome identifier UP000002311; downloaded 21 December 2018), along with 110 common laboratory contaminants (thegpm.org/crap/) and the E. coli PPK protein sequence, and all of these sequences were reversed for a decoy database search. A database search was performed on MS/MS spectra from precursor ions with a charge state of 4 at most. Trypsin was set as the digestion enzyme. Using an X!Tandem two-pass search, the initial search was performed with one miscleavage considered, and a refinement search looked for additional unanticipated miscleavages in peptides from proteins identified in the first pass. Fragment ion and parent ion mass tolerances were set to 20 ppm. Single-amino-acid polymorphisms were checked for each peptide residue in the first search, using a default list of polymorphisms from the single nucleotide polymorphism (SNP) annotations in ENSEMBL (71). Carbamidomethylations of cysteine and selenocysteine were specified as fixed modifications. Variable modifications included were glutamic acid→pyroglutamic acid of the N terminus, glutamine→pyroglutamine of the N terminus, ammonia loss of the N terminus, deamidation of asparagine and glutamine, oxidation and dioxidation of both methionine and tryptophan, and N-terminal acetylation. Validation of peptide and protein identifications was done using the Scaffold program (version 4.11.1; Proteome Software, Inc., Portland, OR). The ProteinProphet algorithm was used to assign protein identification probabilities. Peptide identifications associated with an FDR of <1% were deemed of high confidence. Protein identifications with an FDR of <1% and associated with at least 2 high-confidence peptides were then used in all downstream analyses. Proteins were grouped into protein groups if they shared significant peptide evidence. To satisfy the principles of parsimony, proteins sharing the same peptides were grouped if it was not possible to differentiate them based on MS/MS analyses.

### Differential protein expression analysis.

Protein and protein group spectral counts were retrieved from Scaffold. To account for missing values, spectral counts were imputed for all spectral count values equal to zero by randomly sampling spectral count values from the bottom 20% of nonzero spectral counts. Spectral counts were normalized by the total number of spectral counts in each MS/MS run. Protein differential expression was assessed using two-tailed, two-sample Student’s *t* test assuming unequal variance on normalized spectral counts. Differential expression was assessed using a *t* test only for proteins that were reproducibly identified in 3 or more replicates under both experimental conditions. The Benjamini-Hochberg procedure (72) was used to correct for multiple-hypothesis testing. Proteins were deemed differentially expressed when their FDR-adjusted *P* value was ≤0.1. In addition, proteins with extreme expression differences (i.e., detected in all replicates under one condition and in none under the other condition) are also reported and named “all-or-none” proteins.

### Gene Ontology enrichment analysis.

In order to investigate key functional changes in response to the ectopic expression of PPK, a Gene Ontology (73) enrichment analysis was performed using Ontologizer (74). Differentially expressed proteins (FDR-adjusted *P* value of ≤0.1) and all-or-none proteins were evaluated for enrichment against a background of all identified proteins in all MS/MS runs (FDR of <1%). The OBO ontology file and GAF annotation file used for Gene Ontology enrichment analysis were downloaded from the Gene Ontology database (http://geneontology.org/) on 22 August 2019 and 20 January 2020, respectively. The *P* values were adjusted using the Benjamini-Hochberg procedure to correct for multiple-hypothesis testing.

### Protein-protein interaction network.

The STRING (23) database was used to construct a protein-protein interaction network between differentially expressed proteins (FDR-adjusted *P* value of ≤0.1) and all-or-none proteins. The network was constructed using the STRING medium-confidence (0.4) threshold for experimental evidence and database evidence of interactions. Proteins without any protein-protein interactions were removed from the network.

### YEASTRACT analysis.

Genes of differentially expressed proteins (FDR-adjusted *P* value of ≤0.1) and all-or-none proteins were ranked by transcription factor using YEASTRACT (http://www.yeastract.com/formrankbytf.php) (30). Documented regulations were filtered by DNA binding or expression evidence, and transcription factors acting as an activator or inhibitor and all transcription factors were checked.

### Immunoprecipitation.

Cells were diluted to an OD_600_ of 0.2 in YEP medium with 2% raffinose and grown for 3 h before inducing 3HA-Ddp1 expression with 2% galactose for 2 h. Yeast cell pellets of approximately 50 OD_600_ units were resuspended in 750 μL of lysis buffer (0.1% NP-40, 150 mM NaCl, 50 mM Tris-HCl [pH 8], 10% glycerol); 15 mL of buffer was supplemented with 30 μL of 1 M imidazole, 15 μL of 1 M sodium fluoride, 17.25 μL of 1 M sodium molybdate, 75 μL of 200 mM sodium orthovanadate, 75 μL of 1 M dithiothreitol (DTT), 15 μL of 5 mM cantharidin, 150 μL of 2.5 mM (−)-*p*-bromotetramisole oxalate, 75 μL of 1 M glycerol-2-phosphate, 15 μL of 0.1 M phenylmethylsulfonyl fluoride (PMSF), and a 15-mL Roche cOmplete protease inhibitor tablet with EDTA (15, 51); and 2-mL screw-cap tubes were filled with acid-washed glass beads. Cells were lysed with 8 1.5-min pulses on a BioSpec minibeadbeater with incubation on ice between pulses. Lysates were clarified by centrifugation at 15,000 × *g* for 10 min at 4°C. The supernatant was collected and centrifuged again at 15,000 × *g* for 10 min at 4°C. Anti-HA antibody was added to each lysate at a dilution of 1/650, and the mixture was rotated end over end for 1 h at 4°C. After incubation, 20 μL of protein A magnetic beads was washed 3 times with 700 μL of lysis buffer and added to each sample, and the samples were rotated end over end for 1 h at 4°C. The samples were centrifuged briefly to pellet the beads, and the lysate was removed. The beads were washed 3 times with 700 μL of lysis buffer and eluted with sample buffer by boiling for 10 min.

### Immunoblotting.

Proteins were extracted using a method that was described previously (15). That method is described again here for convenience. Cell pellets at an OD_600_ of 3 to 6 (measured using an Eppendorf BioPhotometer) were resuspended in 300 μL of a solution containing 20% trichloroacetic acid (TCA) and 100 μL of acid-washed glass beads. Cells were lysed with 2 3-min pulses on a BioSpec minibeadbeater. The supernatant was removed and kept. Beads were washed with 300 μL 5% TCA, and this supernatant was combined with the first 300 μL. This mixture was clarified via centrifugation at 16,000 × *g* for 4 min at 4°C. The resulting pellet was dissolved in 100 μL of SDS-PAGE loading buffer supplemented with a 1/10 volume of 1.5 M Tris-HCl (pH 8.8) and a 1/10 volume of 1 M DTT and boiled for 8 min. The samples were centrifuged again at 16,000 × *g* for 4 min, and the supernatant was recovered. Approximately 10 to 20 μL was loaded per gel (corresponding to about 75 μg of protein). Unless stated otherwise in the figure legends, protein samples were run on 10% SDS-PAGE gels made from a 30% acrylamide/bis-acrylamide (37.5:1). To resolve polyphosphorylation-induced shifts, protein samples were run on NuPAGE 4 to 12% bis-Tris gels. Buffers used for both gel types were described previously (15). All proteins were transferred to polyvinylidene difluoride (PVDF) membranes before probing with antibodies. The antibodies used for this work are detailed in Table S4.

10.1128/mbio.00390-22.9 TABLE S4 Antibodies used in this work. Download Table S4, XLSX file, 0.01 MB. Copyright © 2022 McCarthy et al. 2022 McCarthy et al. https://creativecommons.org/licenses/by/4.0/ This content is distributed under the terms of the Creative Commons Attribution 4.0 International license.

### Spot tests.

Cells from freshly grown plates were diluted in water, and 5- or 10-fold serial dilutions were spotted onto the indicated media and allowed to dry prior to incubation at 30°C for 2 to 3 days.

### Liquid growth curves.

Cultures grown overnight in YEP medium with 2% raffinose were diluted to an OD_600_ of 0.2 in YEP medium with 2% raffinose and incubated at 30°C with shaking for 4.5 h (OD_600_ = 0.6 to 0.8). Cultures were subsequently diluted to an OD_600_ of 0.1 (measured using an Eppendorf BioPhotometer) in YEP medium with 2% raffinose or YEP medium with 2% galactose. Two hundred microliters of cells was pipetted into the wells of a 100-well honeycomb plate, and the plate was incubated in a Bioscreen C plate reader (Oy Growth Curves) set at 30°C with continuous shaking. A wavelength of 600 nm was used to measure the optical density every 15 min for 24 h.

### RT-qPCR analysis. (i) RNA extraction.

Cell pellets of 12 OD_600_ units were resuspended in a solution containing 1 mL of cold TRIzol and 200 μL of acid-washed glass beads. Cells were lysed with 6 30-s pulses on a BioSpec minibeadbeater with 2 min on ice between cycles. Two hundred microliters of chloroform was added, and the samples were vortexed for 20 s and incubated at room temperature for 5 min. The samples were centrifuged at 16,000 × *g* for 15 min at 4°C. The aqueous phase was transferred to a fresh screw-cap tube containing 400 μL of chloroform, and the extraction step was repeated as described above. The aqueous phase was transferred to a fresh 1.5-mL Eppendorf tube, RNA was precipitated by adding 500 μL (1:1 volume) of cold isopropanol and 1 μL of Glycoblue, and the mixture was incubated for 30 min at −20°C. RNA was pelleted by centrifugation for 20 min at 20,000 × *g* at 4°C, and the supernatant was discarded. The pellet was washed with 1 mL of cold 70% ethanol and centrifuged at 20,000 × *g* for 10 min at 4°C. The ethanol was discarded, and the pellets were allowed to air dry for 10 min in a fume hood. The pellets were dissolved in 30 μL of RNase-free water and heated at 55°C for 5 min to ensure resolubilization of the RNA. The quality of the RNA was analyzed by nanodrop and agarose gel methods. Extracted RNA was DNase treated according to the Invitrogen Ambion DNase I (RNase-free) protocol. DNase was removed by phenol-chloroform extraction; a 1:1 volume of a phenol-chloroform-isoamyl solution (25:24:1) was added, and the samples were vortexed for 2 min. The samples were centrifuged at 20,000 × *g* for 10 min at 4°C. The aqueous phase was transferred to an Eppendorf tube containing 1 mL of cold ethanol and 16 μL of sodium acetate, and RNA was left to precipitate overnight at −20°C. RNA was pelleted by centrifugation for 20 min at 20,000 × *g* at 4°C, and the supernatant was discarded. The pellet was washed with 1 mL of cold 70% ethanol and centrifuged at 20,000 × *g* for 10 min at 4°C. The ethanol was discarded, and the pellets were allowed to air dry for 10 min in a fume hood. The pellets were dissolved in 25 μL of RNase-free water and heated at 55°C for 5 min.

### (ii) cDNA synthesis and qPCR.

One microgram of RNA was reverse transcribed to cDNA using 5× All-In-One reverse transcription (RT) master mix (Applied Biological Materials). Reaction mixtures were incubated in a thermocycler at 37°C for 15 min, 25°C for 10 min, 60°C for 50 min, and 85°C for 5 min. cDNA was diluted 1:5 with RNase-free water, aliquoted into 20-μL working solutions, and stored at −80°C. Quantitative PCRs (qPCRs) were carried out using iQ SYBR green supermix according to the manufacturer’s protocol under the following conditions: 95°C for 3 min and 40 cycles of 95°C for 15 s, 60°C for 30 s, and 72°C for 30 s. Melt curve analysis was performed at the end of each run, and standard curves using genomic DNA were performed to assess the primer efficiency for each primer pair. All qPCRs were completed in 3 technical replicates with 5 biological replicates. Changes in mRNA expression were calculated using the ΔΔ*C_T_* method, and statistical analysis was performed using Bio-Rad CFX Maestro 2.3 version 5.3.022.1030 software, which uses unpaired two-tailed Student’s *t* test based on the geometric mean. Significance was also determined using standard unpaired two-tailed Student’s *t* test performed using GraphPad Prism version 9. The primers used are listed in Table S5B.

### Data availability.

The mass spectrometry proteomics data have been deposited to the ProteomeXchange Consortium via the PRIDE (75) partner repository with the data set identifier PXD031326.
