# Supplementary figures and images for: Ddp1 Cooperates with Ppx1 to Counter a Stress Response Initiated by Nonvacuolar Polyphosphate
Source: mBio. 2022 Jul 7;13(4):e00390-22. doi: 10.1128/mbio.00390-22 (PMC9426566; doi:10.1128/mbio.00390-22)

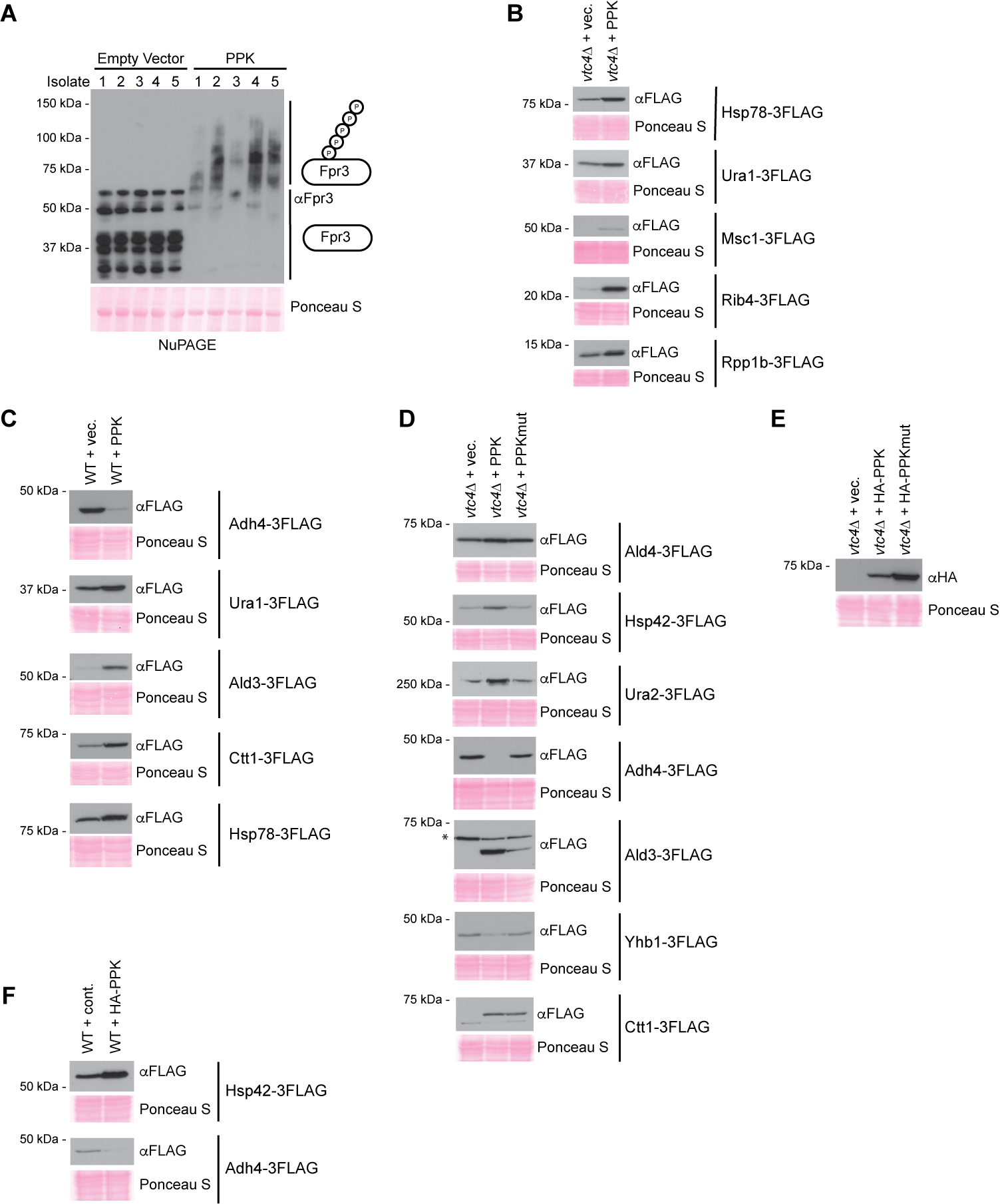

Supplement: FIG S1 [file mbio.00390-22-s0001.tif]

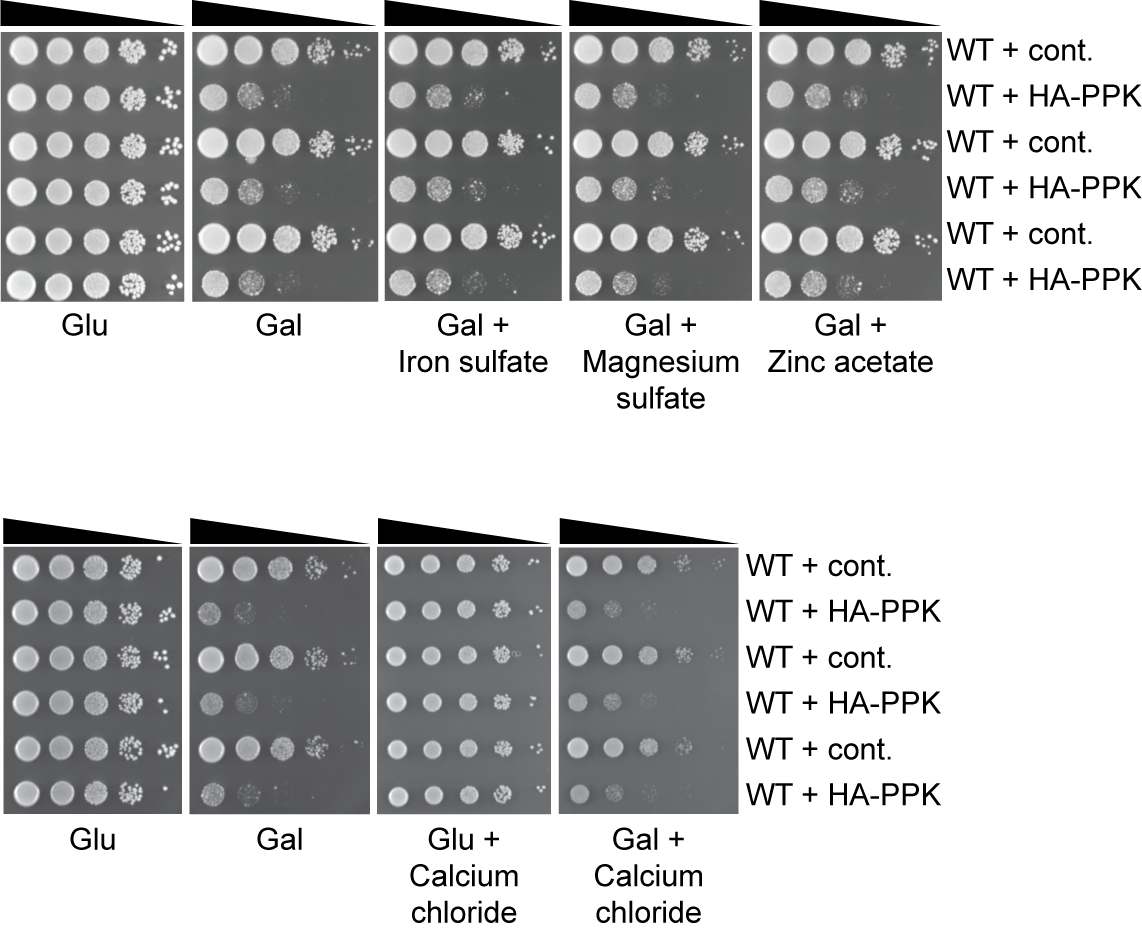

Supplement: FIG S2 [file mbio.00390-22-s0002.tif]

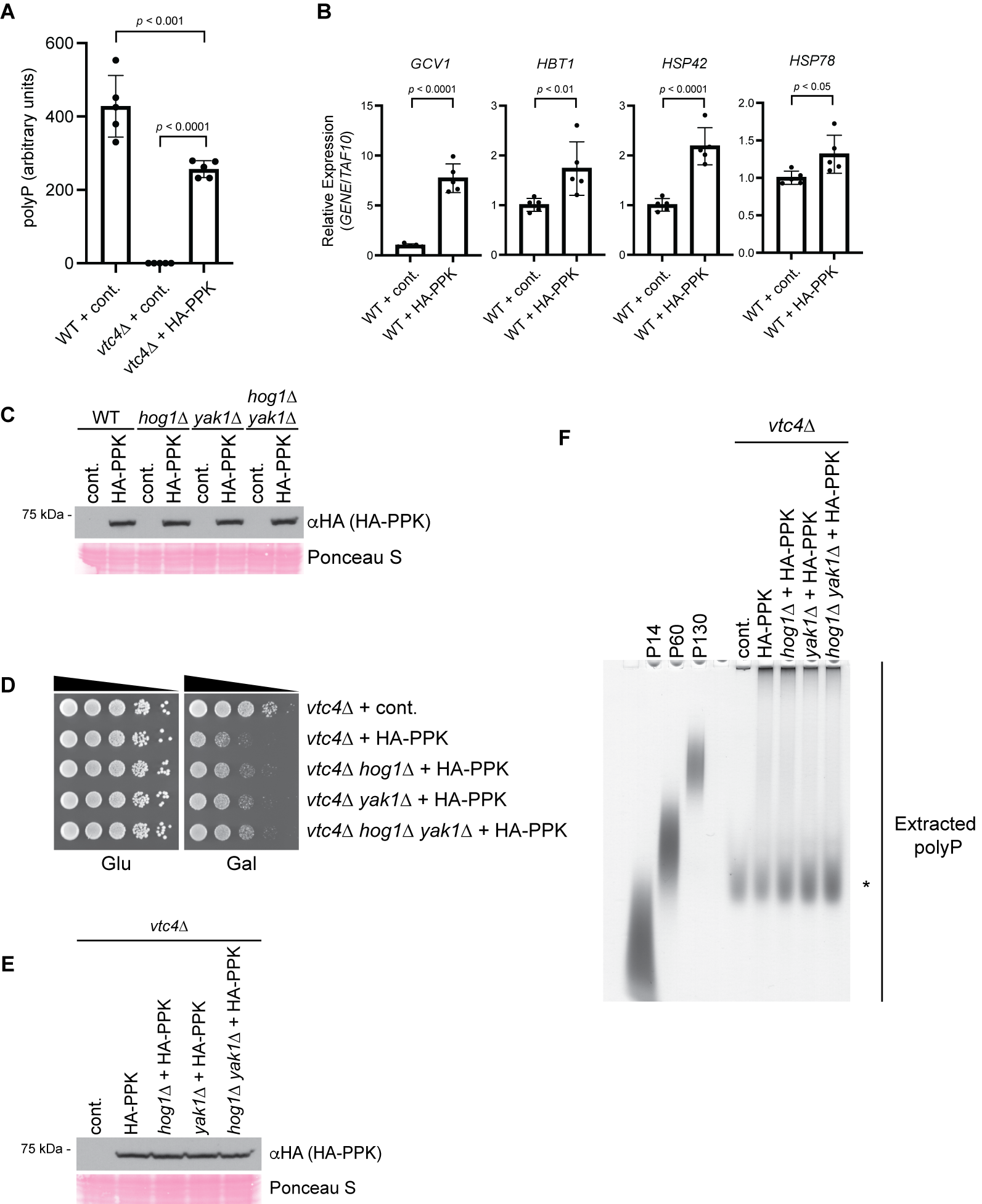

Supplement: FIG S3 [file mbio.00390-22-s0003.tif]

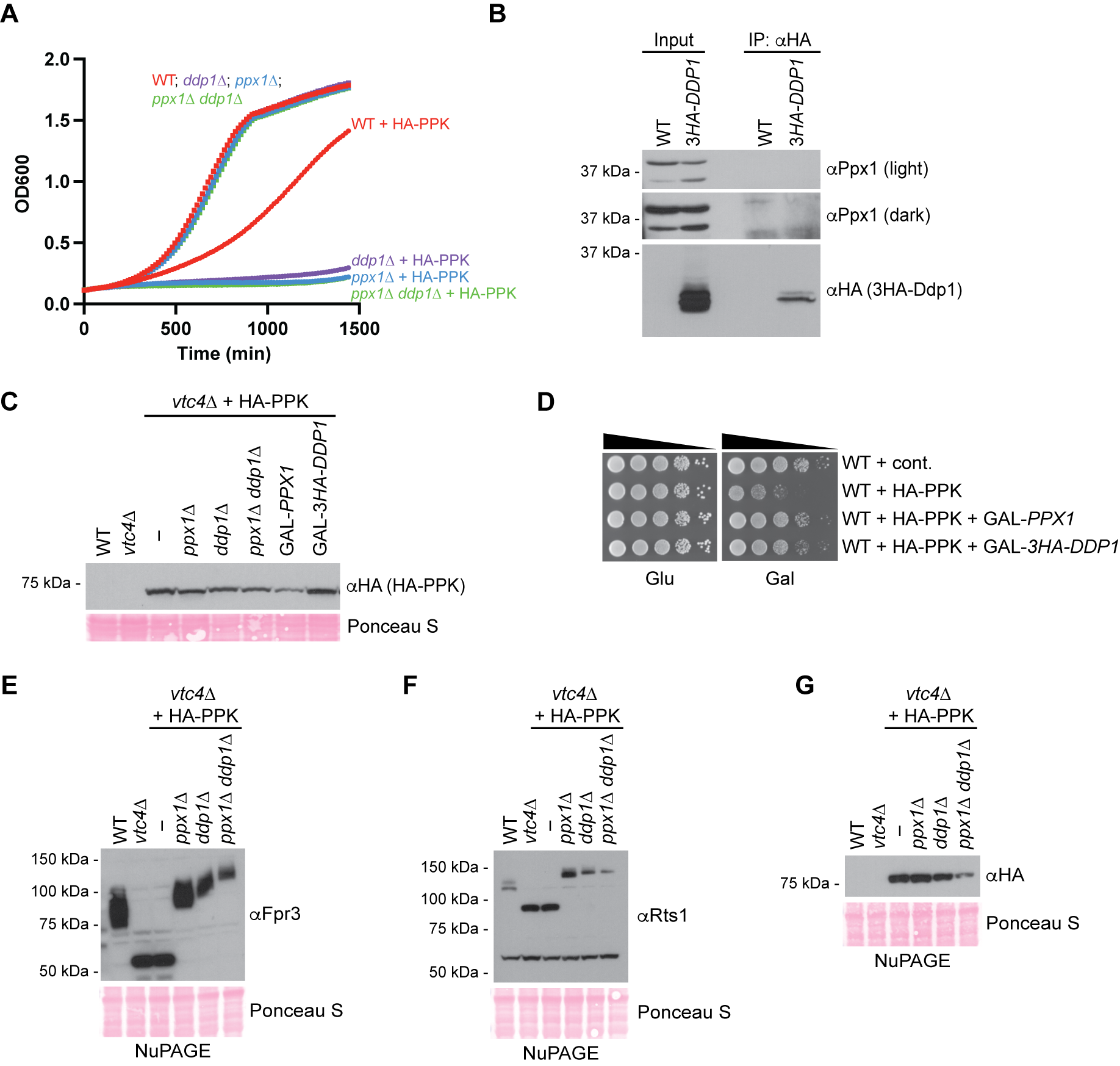

Supplement: FIG S4 [file mbio.00390-22-s0004.tif]

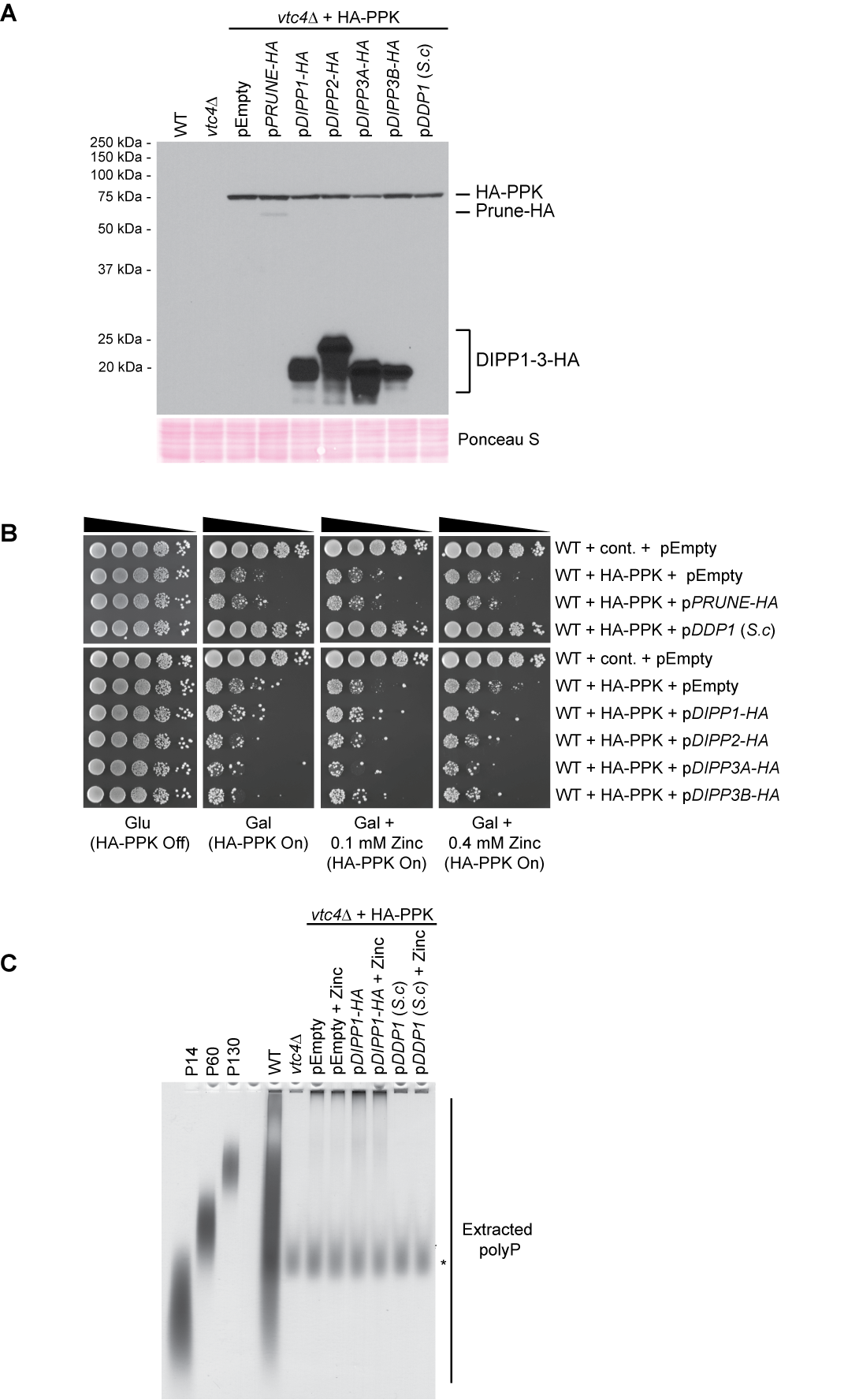

Supplement: FIG S5 [file mbio.00390-22-s0005.tif]
